# Construction of a classification model for dementia among Brazilian adults aged 50 and over

**DOI:** 10.3389/fnagi.2026.1789012

**Published:** 2026-04-15

**Authors:** Felipe da Silva Menezes, Maria Clara Falcão Guerra Barretto, Elliot Quinten Crispiniano Garcia, Tiago Alessandro Espinola Ferreira, Joao Guilherme Bezerra Alves

**Affiliations:** 1 Instituto de Medicina Integral Professor Fernando Figueira (IMIP), Recife, Brazil; 2 Futuro Tech, Recife, Brazil; 3 Universidade Federal Rural de Pernambuco (UFRPE), Recife, Brazil

**Keywords:** Brazil, cognitive dysfunction, health of the elderly, random forest, risk factors

## Abstract

**Background:**

Dementia is a multifactorial and debilitating condition marked by cognitive decline and behavioral changes that compromise independence and daily activities. This condition is a growing challenge in Brazil, and early identification of associated factors can guide preventive strategies and health policies.

**Objectives:**

To build a dementia classification model for middle-aged and older adults Brazilians combining variable selection and multivariable analysis, using low-cost variables, including variables potentially modifiable and non-modifiable sociodemographic variables.

**Methods:**

Observational study employed a cross-sectional design and a classification modeling approach to estimate probable dementia and analyze the odds of dementia, using data from the Brazilian Longitudinal Study of Aging, involving 9,412 participants. Dementia was determined based on neuropsychological assessment and informant-based cognitive function. Analyses were performed with Random Forest (RF) and multivariable Logistic Regression (LR).

**Results:**

The prevalence of dementia was 9.6%. The highest odds of dementia were observed in illiterate individuals (Odds Ratio (OR) = 7.42; 95% Confidence Interval (CI): 4.04–13.62), individuals aged 90 years or older (OR = 11.00; 95% CI: 5.05–23.95), low weight (OR = 2.11; 95% CI: 1.12–3.97), low handgrip strength (OR = 2.50; 95% CI: 1.09–5.76), self-reported black skin color (OR = 1.47; 95% CI: 1.07–2.00), physical inactivity (OR = 1.61; 95% CI: 1.25–2.08), self-reported hearing loss (OR = 1.65; 95% CI: 1.16–2.37), and presence of depressive symptoms (OR = 1.72; 95% CI: 1.36–2.16). In contrast, higher education (OR = 0.44; 95% CI: 0.21–0.94), greater life satisfaction (OR = 0.72; 95% CI: 0.52–0.99), and being employed (OR = 0.78; 95% CI: 0.61–1.00) were protective factors. The RF model outperformed LR, achieving an area under the ROC curve of 0.776 (95% CI: 0.740–0.811), with sensitivity of 0.708, specificity of 0.702, precision of 0.201, Precision-Recall Area Under the Curve (PR-AUC) of 0.261 (95% CI: 0.217–0.319), F1-score of 0.311, G-means of 0.705, and accuracy of 0.703.

**Conclusion:**

The findings reinforce the multidimensional nature of dementia and the importance of accessible factors for supporting screening/triage and prioritization in primary care. Strengthening public policies focused on promoting brain health can contribute significantly to the efficient allocation of resources in primary care and dementia prevention in Brazil.

## 1 Introduction

Dementia is defined by the World Health Organization (WHO) (World Health Organization, 2026) as a progressive neurological condition characterized by impairment in multiple cognitive functions, including memory, reasoning, language, and the ability to perform everyday activities, exceeding the decline expected from normal aging. It is a clinical syndrome that may be triggered by various neurodegenerative pathologies, ultimately leading to the gradual and irreversible loss of nerve cells and to structural and functional brain impairment. Cognitive deterioration is often accompanied by behavioral, emotional, and motivational changes, which substantially affect individual autonomy and quality of life. The impact of dementia extends beyond the affected individual, generating physical, emotional, social, and economic consequences for families, caregivers, and healthcare systems.

In Brazil, the Ministry of Health estimates that approximately two million Brazilians are living with some form of dementia. However, preliminary data indicate that over 70% of those affected remain undiagnosed, which further heightens concern (Ministério da Saúde, 2026), especially considering that recent estimates show an overall dementia prevalence of 5.8% among people aged 60 and older. Prevalence is higher among women (6.8%) than men (4.6%), ranging from 3.2% in the 60–64 age group to 42.8% among individuals aged 90 and above, and is more prevalent among illiterate individuals (16.5%) compared to those with a university-level education or higher (2.1%) (Bertola et al., 2023).

A transitional state between normal cognitive functioning and dementia is known as cognitive impairment, which is characterized by a decline greater than expected for a person’s age and educational level. It affects memory, attention, language, and reasoning abilities, but does not significantly interfere with daily activities (Anand and Schoo, 2024). Estimates indicate a prevalence of 8.1% among individuals aged 60 and older. As with dementia, cognitive impairment is less common in men (6.8%) than in women (9.1%). It’s prevalence remains stable across age groups from 60 to 79 but increases to 11.8 and 12% in the 80–84 and 85–89 age groups, respectively. Interestingly, it decreases to 10% among those aged 90 and over. Regarding educational level, prevalence ranges from 9.4% among illiterate individuals to 10.8% among those with a university-level education or higher (Bertola et al., 2023).

In recent years, efforts have been made to identify potential risk factors and early intervention strategies aimed at reducing the likelihood of dementia onset and delaying it’s progression. It is plausible to assume that anticipating action regarding certain modifiable risk factors could postpone the onset of the disease, given that dementia has prodromal characteristics and may begin to manifest decades before clinical symptoms appear (Oliveira et al., 2019). In a global context, it is vital to consider the significant rise in dementia incidence in Low- and Middle-Income Countries (LMICs) compared to High-Income Countries (HICs) (Livingston et al., 2024). The early and accurate identification of individuals at high risk for dementia plays a crucial role in the effective implementation of preventive strategies.

It is imperative to have an assessment that is clinically feasible for identifying individuals at high risk, especially considering that in Latin American countries such as Brazil, there are significant challenges related to dementia development indicators (Livingston et al., 2024). These challenges are reflected in key characteristics, such as: (i) low income; and (ii) high levels of social inequality (Livingston et al., 2024). These socioeconomic factors often pose barriers to accessing high-cost diagnostic tools such as neuroimaging or biomarkers, which directly affects early detection and effective monitoring. In light of these limitations, there is a pressing need to consider alternative approaches, using accessible variables for both clinical use and large-scale community-based studies.

The current epidemiological context demands a strong prioritization of dementia across all levels, from local settings to the global stage (Avan and Hachinski, 2022). Identifying modifiable factors that can be addressed in advance helps to promote brain health, especially considering that approximately 45% of dementia cases could be delayed or prevented through risk factor modification (Livingston et al., 2024). This underscores the urgent need for innovation and research in monitoring these variables (United Nations, 2023; World Health Organization, 2023). Evidence shows that machine learning algorithms outperform traditional models in predictive performance within medical contexts, due to their ability to manage complex and non-linear relationships. However, traditional models offer advantages such as transparency and interpretability, which are essential in clinical research settings (Couronné et al., 2018; Goldstein et al., 2017; Xi et al., 2022). In this regard, combining both approaches allows us to leverage the strengths of each when it comes to predicting health outcomes.

Currently, a number of models have been developed for predicting cognitive impairment and dementia (Gao et al., 2022; Licher et al., 2018; Schiepers et al., 2018; Zhang et al., 2024), particularly using accessible variables that encompass demographic, socioeconomic, cognitive, functional, and physical factors (Wang et al., 2025). However, it is important to highlight that all of these models were developed using data from populations in foreign countries, which may present a significant limitation when generalizing findings to the Brazilian population. This underscores the need for comparative studies or detailed analyses to assess their effectiveness and relevance within the Brazilian context. Furthermore, a Brazilian study (Szlejf et al., 2023) developed a predictive model for cognitive impairment based on accessible variables in primary care, using a machine learning approach. While this contribution is significant, it is important to note that the data used are not representative of the broader middle-aged and older adult population in Brazil, as the sample was composed mainly of civil servants, who generally have higher levels of education and income compared to the national average. This limitation may affect the generalizability of the results to the broader Brazilian population, particularly in relation to social inequalities and disparities in access to healthcare.

Given this scenario, there is a clear need to develop classification model for dementia using data that are representative of the Brazilian middle-aged and older adult population, specifically taking into account sociodemographic diversity and challenges related to access to high-cost diagnostic exams. The use of accessible, low-cost variables combined with innovative approaches—such as machine learning integrated with traditional statistical analysis—offers a meaningful contribution to the early identification of individuals at risk, as well as to more effective and targeted interventions in primary care settings.

In this context, the present study aims to address a gap in the national literature by building a dementia classification model for Brazilian middle-aged and older adults. The model is implemented in Python and combines variable selection using Random Forest (RF) algorithm with multivariable analysis using Logistic Regression (LR), analyzing low-cost and variables potentially modifiable (lifestyle/clinical/psychosocial) and non-modifiable sociodemographic variables associated with the outcome.

## 2 Materials and methods

### 2.1 Study characteristics

This is an observational cross-sectional study using a classification modeling approach to identify probable dementia based on sociodemographic and health-related variables. The Brazilian Longitudinal Study of Aging (ELSI-Brazil) was funded by the Brazilian Ministry of Health: DECIT/SCTIE—Department of Science and Technology of the Secretariat of Science, Technology and Strategic Inputs (Grants: 404965/2012-1 and TED 28/2017); and COPID/DECIV/SAPS—Coordination of Health for the Older Adult in Primary Care, Department of Life Cycles of the Secretariat of Primary Health Care (Grants: 20836, 22566, 23700, 25560, 25552, and 27510). Ethical approval was granted by the FIOCRUZ Ethics Committee in Minas Gerais, and the study was registered on the *Plataforma Brasil* (CAAE: 34649814.3.0000.5091). All participants signed an informed consent form prior to data collection. This study was also approved by the Research Ethics Committee of the Instituto de Medicina Integral Professor Fernando Figueira (CEP-IMIP), under CAAE: 88342525.5.0000.5201. The study was conducted in accordance with the principles of the Declaration of Helsinki, the guidelines of Resolution 466/12 of the Brazilian National Health Council, and the General Data Protection Law (Law No. 13.709, of August 14, 2018).

### 2.2 Eligibility criteria and data collection procedure

#### 2.2.1 Inclusion criteria

- All individuals aged 50 years or older, non-institutionalized, residing in Brazil, who participated in the individual interview during the first wave.

#### 2.2.2 Exclusion criteria

- Incomplete data from the cognitive test battery or informant-reported cognitive function;
- Variables are not relevant to the objectives of the study.

### 2.3 Data collection and study population

The data were obtained from the ELSI-Brazil database, a population-based cohort study with a meticulously planned sampling strategy designed to accurately reflect the diversity of the non-institutionalized population aged 50 years and older. The sample includes individuals from both urban and rural areas across municipalities of varying sizes in Brazil, with data collection conducted between 2015 and 2016 (ELSI Brasil, 2025; Lima-Costa et al., 2018). The final sample consisted of 9,412 participants residing in 70 municipalities from different geographic regions. A complex, multi-stage sampling design was used, involving stratification of primary sampling units (municipalities), census tracts, and households. Municipalities were grouped into four strata based on population size. In the first three strata, sampling was conducted in three stages: selection of municipalities, census tracts, and households. In the fourth stratum, which included the largest municipalities, sampling was conducted in two stages: selection of census tracts and households (ELSI Brasil, 2025; Lima-Costa et al., 2018). All residents aged 50 years or older in the selected households were eligible for interviews and physical measurements.

### 2.4 Research tools

#### 2.4.1 Cognitive function

For the dependent variable, a participant classification approach was used to identify cognitive status within the sample, distinguishing between normal cognition, cognitive impairment, and dementia. This classification was based on a combination of cognitive performance and functional capacity in Instrumental Activities of Daily Living (IADLs) (Bertola et al., 2023).

To assess cognitive performance, a battery of neuropsychological tests was used to evaluate different cognitive domains, namely: temporal orientation, semantic verbal fluency, a 10-word list test for immediate and delayed recall, prospective memory, and semantic memory. A detailed description of all subdomains of the neuropsychological test battery can be found in Supplementary Table 1.

Initially, the numerical results from each test subdomain were used to generate raw scores, such as the number of correct answers and/or performance rankings on the test (Piccininni et al., 2022). For the questions related to temporal orientation (day, month, year, and day of the week) and semantic memory (naming certain objects and political figures), one point was assigned for each correct answer. These were then summed to produce a raw score for each participant. For the subdomains of semantic verbal fluency and the 10-word list for immediate and delayed recall, the number of words (animals) mentioned, and the number of words recalled (immediately and after a delay), respectively, were recorded. Prospective memory was assessed using a scoring system ranging from 0 to 5, with the correct execution of the requested task receiving the highest score.

Next, raw scores were standardized using z-score calculation, which involved subtracting each individual’s score from the sample mean and dividing the result by the sample standard deviation. This yielded a standardized score for each participant, allowing the results to reflect deviations from the mean and to be comparable across different subdomains. The meaning of the standardized scores from all subdomains was then used to compute a global cognitive score.

#### 2.4.2 Informant-based cognitive function

For participants unable to complete the cognitive test battery, the short version of the *Informant Questionnaire on Cognitive Decline in the Elderly* (IQCODE), consisting of 16 items and validated in Brazil (Perroco et al., 2008; Sanchez and Lourenço, 2009; Sanchez and Lourenço, 2013), was administered. Based on the results, individuals were classified as having normal cognition, cognitive impairment, or dementia.

The IQCODE is the instrument used by ELSI-Brazil to assess cognitive function based on information provided by someone close to the participant, such as a family member or caregiver. It aims to identify cognitive changes, particularly in cases where the individual may be unable to accurately report their own condition.

This section (*Cognitive function for a proxy respondent*) consisted of questions directed at the informant, who was asked to compare the participant’s current memory and cognitive abilities with their condition 2 years prior. The questions were structured to address various aspects of memory and cognition, with responses categorized into pre-established options. The informant was instructed to respond based on their perception of the degree of improvement, stability, or decline.

Responses were collected using standardized options such as “Improved,” “Did not change much,” or “Worsened,” along with subcategories to indicate the intensity of the changes, such as “Improved a lot,” “Some improvement,” “Some decline,” or “Worsened a lot.” The options “Don’t know/Did not respond” were used in cases where the informant lacked sufficient information to answer, and these were treated as missing data and excluded from the analysis. All 16 items of the short version of the IQCODE are detailed in Supplementary Table 2.

Each of the 16 IQCODE items reflects the degree of perceived change, rated using a Likert scale as follows: (1) improved a lot, (2) some improvement, (3) did not change much, (4) some decline, and (5) worsened a lot. The final score is calculated as the average of all valid responses—i.e., only the items that were answered —allowing for the handling of missing data. Participants with scores below 3.22 are categorized as having normal cognition, as this indicates no significant cognitive decline. Those scoring between 3.22 and 3.47 are considered to have cognitive impairment, suggesting a mild perceived decline not sufficient to be classified as dementia. Lastly, participants with scores equal to or > 3.48 are classified as having dementia, reflecting a higher degree of cognitive deterioration as perceived by the informant (Carrabba et al., 2015; Perroco et al., 2008; Sanchez and Lourenço, 2009; Sanchez and Lourenço, 2013).

#### 2.4.3 Neuropsychological test norms

To calculate the z-scores that comprise the global cognitive score, it is first necessary to establish correction factors or norms based on the raw scores of a normative subsample. The purpose is to provide regression-based adjusted norms (Manly et al., 2005) for evaluating the cognitive performance of the full sample. This normative subsample consists of “healthy” individuals without diseases or conditions that could lead to cognitive or behavioral impairments affecting performance on the cognitive test battery.

Full details of each variable used to define the normative subsample are provided in Supplementary Table 3. Participants presenting the following conditions were excluded from the normative subsample:

- Self-reported visual and hearing impairments;
- Self-reported medical diagnosis of depression or depressive symptoms assessed using the eight-item Center for Epidemiologic Studies Depression Scale (CES-D8), with a cutoff score of 4 (Batistoni et al., 2025; Dang et al., 2020);
- Self-reported history of diagnosis of stroke, Alzheimer’s disease, or Parkinson’s disease;
- Excessive alcohol consumption based on the criteria of the National Institute on Alcohol Abuse and Alcoholism (NIAAA), defined as weekly intake of 14 drinks or daily intake of 4 drinks for men, and weekly intake of 7 drinks or daily intake of 3 drinks for women (Alcohol’s Effects on Health, 2025; Devos-Comby and Lange, 2008; Kerr and Stockwell, 2012);
- Self-reported memory complaints or those reported by an informant;
- Self-reported impairment in 4 instrumental activities of daily living, such as managing one’s own finances and medications, using transportation, and using the telephone;
- Missing data and absence of cognitive test results.

A multiple regression analysis was performed using the standardized global cognitive score as the dependent variable and age, sex/gender, and education (in years) as independent variables. Based on the weighted coefficients obtained from the regression model, predicted global cognitive scores were calculated for the entire sample. These predicted scores were then subtracted from the actual scores of each participant, generating regression residuals. The residuals were standardized by dividing them by the standard deviation of the residuals (root mean square error) from the regression performed on the normative subsample. The result was a standardized z-score for each participant’s global cognitive score, which was used to determine the presence of cognitive impairment according to previously established criteria.

#### 2.4.4 Categorization of the response variable

A classification of the participants in the sample was carried out to identify individuals with normal cognition, cognitive impairment, or dementia, based on the evaluation of cognitive function previously described, in combination with assessments of functional capacity. This approach was employed in a prior study using the same database (ELSI-Brazil) (Bertola et al., 2023).

According to the regression-based normative standards, a global cognitive score (z-score) equal to or lower than -1.5 (one and a half standard deviations below the mean) was considered indicative of cognitive impairment. Based on these results, it was possible to determine each individual’s cognitive status, considering both cognitive performance (through the global cognitive z-score) and functional impairment, as assessed through IADLs. Functional impairment was defined by self-reported difficulty in at least four IADLs that are directly related to cognitive function, namely: managing finances, using transportation, using the telephone, and managing medications.

The classification followed these criteria (Carrabba et al., 2015; Perroco et al., 2008; Sanchez and Lourenço, 2009; Sanchez and Lourenço, 2013): (1) Normal cognition was defined as the absence of both cognitive and functional impairment, or the presence of functional impairment not related to cognitive issues (e.g., due to physical limitations), along with an IQCODE score below 3.22; (2) Cognitive impairment was defined as cognitive impairment without functional impairment, or an IQCODE score equal to or > 3.22; (3) Dementia was defined as the presence of both cognitive and functional impairment, or an IQCODE score equal to or > 3.48, for participants who were unable to complete the cognitive assessment and required evaluation via the IQCODE.

##### 2.4.4.1 Sociodemographic characteristics

In this study, we examined six sociodemographic factors: (1) Age—individuals aged 50 years or older, based on their age at the time of the interview, categorized into 5-year age groups; (2) Sex—male and female; (3) Educational level—defined by the highest school grade the individual had successfully completed (never attended school; 1st–8th grade of primary education; 1st–3rd grade of secondary education; adult education/equivalency programs and some college; completed higher education; and postgraduate degrees such as specialization/residency, master’s, or doctoral degrees); (4) Occupational status in the past 30 days; (5) Marital status—single, married/living with partner/in a stable union, divorced or separated, and widowed; (6) Skin color—white, black, brown (mixed race), yellow (East Asian origin, such as Japanese, chinese, korean, etc.), and Indigenous.

Self-reported skin color was included following the classification used in Brazilian national surveys. In the Brazilian context, skin color is primarily understood as a social and structural marker rather than a biological determinant. It’s inclusion in the model was intended to allow the investigation of potential disparities related to social determinants of health, such as unequal access to healthcare services, education, and socioeconomic opportunities (Constante et al., 2021). Including such variables can improve transparency about health inequalities and allow future evaluations of potential differential model performance across population groups, an issue increasingly emphasized in the ethical development of machine learning models in healthcare (Chen et al., 2021).

##### 2.4.4.2 Health and comorbidities

Body Mass Index (BMI) was calculated as the ratio between body weight in kilograms (kg) and height in meters squared (m^2^), using the formula: BMI = weight(kg)/height(m)^2^. To ensure accuracy, measurements were taken with individuals barefoot, standing upright with feet and heels together, and with their back and head against the measuring device. Measurements were taken twice, and the average value was used (Lima-Costa et al., 2018). In accordance with World Health Organization (WHO) guidelines (Zierle-Ghosh and Jan, 2024), BMI was categorized to facilitate data analysis. Although BMI does not directly measure body fat percentage and may overestimate it in highly muscular individuals or underestimate it in those with higher fat mass (Loef and Walach, 2013), it remains widely used for being simple, standardized, low-cost, and quick making it particularly useful in population-based and international studies (Livingston et al., 2024; Veronese et al., 2017).

Blood pressure (BP) was categorized using a cutoff of ≥ 140/90 mm Hg, based on the Brazilian guidelines for hypertension (Malachias et al., 2016), as adopted in previous studies (Aliberti et al., 2021). BP was measured using a validated automatic device (Topouchian et al., 2011), the Omron M3 HEM-7200 (Omron Healthcare Brazil, São Paulo, Brazil). Before measurement, participants were instructed to remain seated and still for at least 5 min. During this time, they were asked not to speak or move, ensuring their blood pressure reached a resting level suitable for accurate measurement. Three measurements were taken, with 2-min intervals between each, and the average BP was calculated (Lima-Costa et al., 2018). BP was then categorized into hypertensive or non-hypertensive.

Diabetes was self-reported and identified using the question: “Has a doctor ever told you that you have diabetes (high blood sugar)?” A “yes” response classified the individual as having diabetes. This self-report approach is widely accepted and has been used in several previous population-based studies (Borelli et al., 2022a; Cochar-Soares et al., 2020; Souza et al., 2024), supporting it is validity and reliability.

High cholesterol was self-reported through the question: “Has a doctor ever told you that you have high cholesterol?” with binary response options (“yes” or “no”). Self-reported high cholesterol emerged as the third most common condition among participants, affecting 30.5% of the sample (Nunes et al., 2018). Including high cholesterol as a variable highly relevant. It’s high prevalence among older adults not only reflects a major public health issue, but may also influence the management of other chronic conditions (Rodrigues et al., 2022; Rodrigues et al., 2023). Furthermore, recent evidence supports it is role as a potentially modifiable risk factor for dementia (Livingston et al., 2024).

Untreated vision loss due to conditions such as cataracts and diabetic retinopathy was collected via self-report in the ELSI-Brazil study. Two questions were used to define cataract status: “Has an ophthalmologist ever told you that you have or had cataracts in one or both eyes?” and “Have you had cataract surgery?” These questions were selected based on evidence showing that individuals with cataracts are at increased risk for all-cause dementia (Kuźma et al., 2021), and that cataract removal is associated with significantly reduced probability of dementia (Lee et al., 2022). In our sample, those who reported a cataract diagnosis but had not undergone surgery were classified as “with cataracts,” while those who had never been diagnosed or who had undergone surgery were classified as “without cataracts.”

Regarding diabetic retinopathy, we used the question: “Has an ophthalmologist ever told you that you have or had diabetic retinopathy (diabetes in the eyes)?” since this condition has been associated with an increased risk of dementia (Lee et al., 2023; Rodrigues et al., 2023). All questions had binary response options (“yes” or “no”). Responses such as “don’t know/did not answer” were treated as missing data. Including these variables may enrich the analysis, as they have emerged as significant and potentially modifiable factors for dementia (Livingston et al., 2024), based on growing evidence (Ferguson et al., 2024; Xiong et al., 2023).

Hearing loss was assessed based on the participant’s own perception of their hearing, including if they used a hearing aid. It was self-reported using the question: “How would you rate your hearing (even when using a hearing aid)?” Response options included: very good or excellent, good, fair, poor, and very poor. Following the approach of a previous study using ELSI-Brazil data, responses were categorized into: good (including “very good or excellent” and “good”), fair, and poor (including “poor” and “very poor”) (Oliveira et al., 2023).

##### 2.4.4.3 Physical activity level, handgrip strength, and lifestyle habits

To assess the level of physical activity, the short version of the *International Physical Activity Questionnaire* (IPAQ) was used (Peixoto et al., 2018), translated and validated for Brazil (Craig et al., 2003; Matsudo et al., 2001) and for use among adults and older individuals (Benedetti et al., 2007). This questionnaire includes questions regarding frequency (days) over the past week and duration (minutes) of activities such as walking (low intensity), moderate, and/or vigorous-intensity exercise. The *Metabolic Equivalent of Task* (MET) was used as a measure to weight each type of activity according to it is energy cost, expressed as a multiple of the resting metabolic rate. A *Total Physical Activity Score* (TPA) in MET-minutes/week was calculated by multiplying the MET score of each activity (3.3 for walking, 4.0 for moderate, and 8.0 for vigorous activity) by the minutes performed and weekly frequency. MET-minutes/week were calculated separately for each activity and then summed. A categorical score was then created based on the number of days, duration, type of activity, and TPA. Cases in which responses included “Don’t know/Did not answer” were treated as missing data and excluded from analysis. All steps followed the *IPAQ Data Processing and Analysis Guidelines* for categorizing physical activity levels (Committee IPAQ, 2025).

Handgrip Strength (HGS) was measured using a hydraulic hand dynamometer with an adjustable handle (SAEHAN^®^, South Korea, JAMAR). The test was conducted three times with a 1-min rest interval between each measurement. Participants chose their dominant hand for the test, which was performed while seated in an armless chair, with the elbow flexed at 90 degrees, the arm alongside the body without support or additional movement during the test, the forearm in a neutral position (thumb pointing up), and the wrist in a comfortable position. The dynamometer handle was placed in the second position or adjusted according to hand size when necessary. During the test, participants were given verbal encouragement as sensory stimuli (de Souza Moreira et al., 2022; dos Santos et al., 2024).

Cut-off points for HGS categorization were established according to international norms (Tomkinson et al., 2024), adjusted by sex and age using percentile distributions of absolute strength (kgf) reported in that study. For individuals aged 50 and older, handgrip strength was classified into five categories: *low* (below the 20th percentile), *slightly low* (20th–39th percentile), *moderate* (40th–59th percentile), *slightly high* (60th–79th percentile), and *high* (80th percentile or higher). Absolute values (in kgf) for men and women are presented in Supplementary Table 4. Responses such as “Refused” or “Did not attempt due to perceived risk” were treated as missing data. For responses like “Attempted but couldn’t” or “Unable,” a score of 0 was assigned, as the HGS test is a functional measure and inability to perform the test reflects compromised health and a complete absence of strength. Furthermore, including HGS in dementia classification models can improve accuracy by incorporating an objective indicator of physical health that reflects both central nervous system integrity and musculoskeletal condition.

Smoking was assessed through self-report using the question: “Do you currently smoke?” The question was presented within a broader context to capture the widest range of smoking behaviors, including manufactured cigarettes, straw cigarettes, or other smoked tobacco products (cigars, cigarillos, pipes, clove or Bali cigarettes, Indian cigarettes or bidis, and hookahs/water pipes). Tobacco products such as snuff, chewing tobacco, and electronic cigarettes were not considered. Response options were: “Yes, daily,” “Yes, less than daily,” “No,” and “Don’t know/Did not answer.” The last option was treated as missing data.

Alcohol consumption was also self-reported based on the following questions: “How many days per week do you usually drink any alcoholic beverage?” and “On a typical day when you drink, how many drinks do you consume?” For the first question, responses ranged from 1 to 7 days, with 0 for individuals who drank less than once a week. The second question referred to the number of drinks per day, with 1 drink equivalent to 1 can of beer, 1 glass of wine, or 1 shot of cachaça, whiskey, or other distilled beverages. Individuals who responded “Don’t know/Did not answer” to either question were excluded as missing data. Excessive alcohol consumption was defined by calculating the total number of drinks per week (drinks per day × days per week), with 21 or more drinks per week (equivalent to 168 grams of pure alcohol) considered excessive (Borelli et al., 2022a; Livingston et al., 2024).

##### 2.4.4.4 Psychosocial factors

Social contact was assessed through questions on social relationships. Three items were used: “How often do you meet in person with any of your children, not counting those who live with you?,” “How often do you meet in person with any of your relatives, not counting those who live with you?,” and “How often do you meet in person with any of your friends, not counting those who live with you?” Possible responses included: “3 or more times per week,” “1 or 2 times per week,” “1 or 2 times per month,” “Every 2 or 3 months,” “Once or twice per year,” and “Less than once a year or never.”

To identify low social contact/social isolation, an individual was considered socially isolated if they reported meeting with children, relatives, or friends less than once a month (Borelli et al., 2022a). Otherwise, participants were categorized as having social contact. If the participant answered “Don’t know/Did not answer” to all three questions, their data were treated as missing and excluded from the social isolation analysis. However, if they responded to at least one of the three items, they were included, since social contact frequency was defined based on the available response(s).

Loneliness was self-reported based on the question: “How often do you feel lonely?” Possible responses were: “Never,” “Sometimes,” and “Always.” Self-reported loneliness was considered present when the participant indicated insufficient social contact according to their own subjective experience. Responses of “Don’t know/Did not answer” were treated as missing data.

Subjective wellbeing (SWB) was evaluated using a life satisfaction ladder. Participants were shown an illustration of a ten-rung ladder representing general life satisfaction, ranging from 1 (lowest level) to 10 (maximum satisfaction). Participants were asked to reflect on their overall SWB and indicate the rung that best represented their current satisfaction. The interviewer recorded the number selected.

Depressive symptoms were assessed using the eight-item version of the Center for Epidemiologic Studies Depression Scale (CES-D8) (Batistoni et al., 2025; Radloff, 1977), a validated short form (Carvalho et al., 2024; Freitas et al., 2023; Lima-Costa et al., 2018). The CES-D8 includes the following items: “During the past week, most of the time, did you feel depressed?,” “During the past week, most of the time, did you feel that everything you did was more difficult than usual?,” “During the past week, most of the time, did you feel that your sleep was not restful, that you woke up not feeling rested?,” “During the past week, most of the time, did you feel happy?,” “During the past week, most of the time, did you feel lonely?,” “During the past week, most of the time, did you enjoy life or feel pleasure from it?,” “During the past week, most of the time, did you feel sad?” and “During the past week, most of the time, did you feel that you couldn’t get going with your activities?.” Each item could be answered with “Yes” or “No.” For items 1, 2, 3, 4, 6, and 8, a “Yes” response scored 1 point. For items 5 and 7, a “No” response scored 1 point. The total score ranges from 0 to 8. A score of 4 or higher was used as the cutoff to indicate the presence of depressive symptoms, while scores below 4 indicated absence of symptoms. Participants who answered “Don’t know/Did not answer” or “Not applicable” were excluded from the analysis of depressive symptoms, and these responses were treated as missing data.

### 2.5 Statistical analysis

The analyses were conducted by the researchers using the Python programming language, version 3.10, through the online platform Google Colaboratory (Google Colab, 2025). The following libraries were used: Numpy version 1.26.4, Pandas version 2.2.2, seaborn version 0.13.2, statsmodels version 0.14.4, scipy version 1.13.1, matplotlib version 3.10.0, and scikit-learn version 1.7.1 (Pedregosa et al., 2011; Perktold et al., 2024; Scikit-Learn, 2025b; Waskom, 2021).

Continuous variables that were not normally distributed (as assessed by the Shapiro–Wilk test) were reported as medians and interquartile ranges; comparisons were performed using the Mann–Whitney U test. Categorical variables were reported as counts and percentages and compared using the chi-square test. For all statistical tests, a *p*-value < 0.05 was considered statistically significant (Chicco et al., 2025).

The application of the RF algorithm enabled the analysis of large volumes of complex data, identifying patterns and combinations between variables that might be overlooked by traditional methods, in addition to it is ability to handle non-linear data (Obermeyer and Emanuel, 2016). Although other feature selection techniques exist, RF was chosen for it is robustness and versatility in handling various types of data. It is particularly effective for binary, imbalanced datasets with multiple variables or complex structures, including correlated variables. Furthermore, a key advantage of this algorithm is it’s intrinsic ability to measure feature importance and use out-of-bag (OOB) error, which provides a reliable estimate of model performance and supports the selection of the optimal number of variables for model construction (Arashi et al., 2022; Drobnič et al., 2020; Li and Mu, 2024; Speiser et al., 2019). The most important features—those with the lowest average error rates selected by the RF algorithm—were subsequently analyzed using multivariable LR.

#### 2.5.1 Data preprocessing

Initially, before proceeding with any steps in this stage, the dataset was split into training (80%) and testing (20%) sets, while preserving the class proportions. All subsequent steps were applied exclusively to the training set, with the test set reserved for later model performance evaluation.

In the training set, missing data were identified for each variable. The K-Nearest Neighbors (KNN) multiple imputation method (Rizvi et al., 2025) was used to replace each missing value with an observed value from similar records in the dataset, identified using a distance metric. In this case, we used the default KNN metric: Euclidean distance, which calculates the square root of the sum of squared differences between non-missing values, assessing similarity between individuals. The closest neighbors were then used to fill in the missing values with the average of their corresponding values. This is crucial in data analysis, as it helps preserve the original data structure and prevents distortion in the distribution of imputed variables (Emmanuel et al., 2021; Ge et al., 2023; Rizvi et al., 2025).

This algorithm is widely applied in health research to address missing data, which can compromise analysis quality, ensuring data integrity and representativeness (Beretta and Santaniello, 2016). In the medical field, KNN imputation improves the accuracy of models and supports decision-making, for example, in heart disease datasets, where this method has shown improved performance, provided a proper balance is maintained between the number of neighbors used and the preservation of the original data structure (Beretta and Santaniello, 2016; Uddin et al., 2022).

Before handling missing values, data normalization was performed. At this point, only the *life satisfaction* variable was normalized using MinMaxScaler, as it is a continuous variable (ranging from 0 to 10). In the KNN algorithm, such variables can disproportionately influence the distance metric and, consequently, affect the selection of nearest neighbors and the imputation of all other variables.

Next, the Elbow Method (Syakur et al., 2018; Wala et al., 2024) was applied to determine the optimal number of neighbors (*k*) for KNN imputation. This technique involves testing different values of *k* and analyzing a graph to identify the point at which increasing *k* no longer results in meaningful performance improvement. This point, known as the “elbow,” corresponds to the ideal value for *k* based on the curve’s shape.

This method was tested on a dataset in which 10% of the values were randomly removed and replaced with NaN (Not a Number), simulating the presence of missing data. This allowed for an evaluation of the imputation method and the determination of the *k-*value that minimizes the Mean Absolute Error (MAE).

The training set (with no missing values) was divided into two subsets to enable comparison between actual and imputed values in the simulated dataset, allowing for MAE calculation. Values of *k* from 1 to 20 were tested to explore different configurations. For each *k*, a KNN imputer was created, and missing values were imputed. MAE was then computed by comparing imputed values with their actual counterparts.

This entire process was repeated 30 times to increase result robustness. The errors from each iteration were stored in a list, enabling the calculation of the average error for each *k-*value. The point at which the error reduction curve shows a significant slowdown, the “elbow,” was selected as the optimal number of neighbors. This approach balances imputation accuracy with model simplicity, allowing for efficient estimation of missing data without unnecessary complexity (Sammouda and El-Zaart, 2021). After determining the optimal *k*, the KNNImputer algorithm (Scikit-Learn, 2025a) was employed to fill in the missing values based on similar individuals in the dataset (nearest neighbors).

#### 2.5.2 Model building

RF (Lebedev et al., 2014; Scikit-Learn, 2026) combines multiple classifiers through parallel decision trees, where each tree is built using a random vector sampled independently and identically distributed for all trees in the forest. At each split node in a tree, a random subset of features is selected to determine the best split. Each tree in the forest produces a classification, and the final decision is made by aggregating the outputs of all trees through majority voting. This method is known as bagging or bootstrap aggregating.

Because the prevalence of dementia in the dataset was relatively low (9.6%), resulting in class imbalance, the RF classifier was implemented using the *class_weight = “balanced”* parameter. This approach automatically adjusts the weight of each class inversely proportional to it is frequency in the training data, increasing the penalty for misclassification of the minority class (dementia). This strategy aims to mitigate bias toward the majority class and improve the model’s ability to identify dementia cases.

Feature importance analysis was based on the reduction in impurity during the construction of decision trees. This process is measured using the Gini index, which quantifies how homogeneous the data are with respect to the target variable (cognitive status). Each time a variable is used to split a node, the decrease in the Gini index is calculated, and the overall importance of a variable is obtained by summing all such reductions across all trees in the forest. The greater this total reduction, the more important the variable is to the model, providing a direct and interpretable estimate of the variable’s relevance to model decisions.

After ranking the variables by importance, we aimed to identify the smallest subset of variables that minimized the out-of-bag (OOB) error, an intrinsic metric of the RF algorithm. OOB error is computed during training using data not selected for building each tree—these forms the OOB sample. For each individual in the OOB sample, classification is made only using trees that did not have access to that individual’s data (by aggregating the votes from these trees). OOB accuracy is then calculated as the proportion of correct classification, and OOB error is defined as 1 minus OOB accuracy.

In this process, variables were added incrementally in order of importance to perform a stepwise RF analysis. Model training began with only the most important variable, followed by the addition of the second most important, and so on, until all variables (in order of decreasing importance) were included. For each subset of variables, training was repeated multiple times using a stratified 10-fold cross-validation approach (Wilimitis and Walsh, 2023) with 30 repetitions, accounting for 300 experiments per iteration.

This technique involves splitting the dataset into multiple subsets, known as folds, allowing the model to be trained repeatedly on different portions of the training set. This approach is essential for estimating the model’s true performance on unseen data, minimizing both bias and variance during training, and ensuring a more robust evaluation.

However, instead of using a separate validation set, we relied on the out-of-bag (OOB) error metric from the RF as the validation method, calculating error on OOB samples. Using this approach, for each additional variable included in the model, we obtained the mean and variability of the OOB error. At the end of this process, we were able to identify the subset of variables that minimized the mean OOB error, thereby determining the most parsimonious and best-performing set of variables (Krstajic et al., 2014).

**FIGURE 1:**
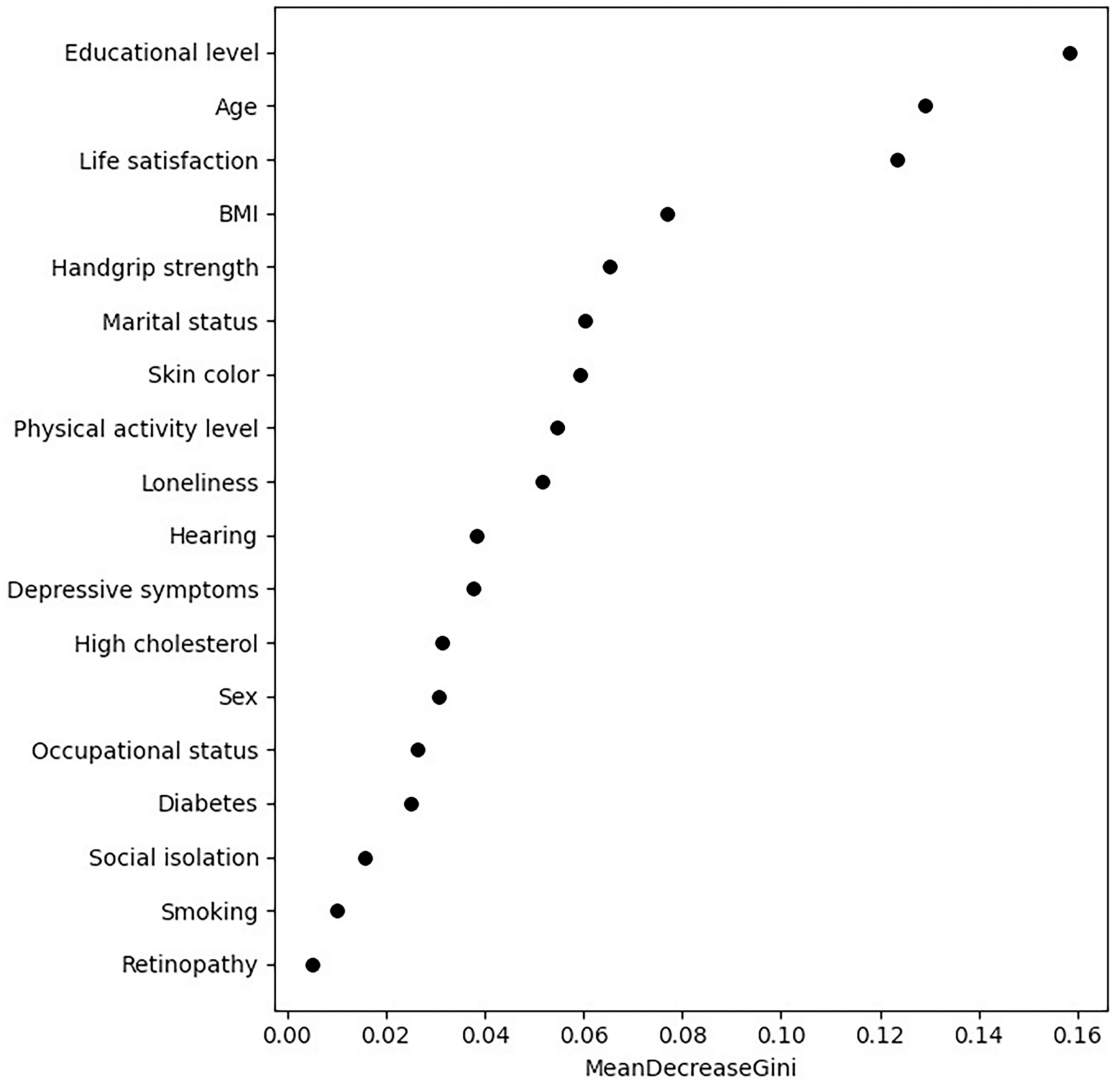
Importance ranking of variables influencing dementia in individuals aged 50 years and older.

To build the final classification model to identify individuals with probable dementia, the variables with the highest importance scores and lowest average OOB error rates were analyzed using multivariable LR, allowing for mutual adjustment between variables and yielding coefficients that were converted into odds ratios (ORs) to enhance model interpretability. Statistical significance was considered at *p* < 0.05. The full model development process is illustrated in Figure 1.

Given the class imbalance in the dataset, evaluation metrics that are sensitive to minority class performance were prioritized. While accuracy provides a general measure of overall correctness, it can be misleading when class distributions are highly skewed. Therefore, additional metrics such as sensitivity (recall) and specificity are essential to assess the model’s ability to correctly identify positive and negative instances, respectively. The inclusion of AUC-ROC further enables the analysis of the model’s discriminative capability across different decision thresholds. Moreover, metrics such as F1-score and G-mean are particularly valuable in imbalanced scenarios, as they emphasize the balance between precision and recall and the balanced performance across classes. Together, the combined use of accuracy, sensitivity, specificity, AUC-ROC, F1-score, and G-mean provides a more robust and reliable assessment of model performance, reducing the risk of biased interpretations and strengthening the overall analysis of the predictive model (Kulatilleke and Samarakoon, 2022; Owusu-Adjei et al., 2023; Salmi et al., 2024).

After performing multiple LR analysis, we identified the variables that were significantly associated with dementia. Using this subset of variables, we retrained the models from scratch on the entire training dataset, employing both RF algorithm and LR for comparison purposes. Finally, we evaluated the performance of both models on the test dataset.

## 3 Results and discussion

### 3.1 Results

#### 3.1.1 General information

Out of all 8.884 participants, 845 had dementia (9.6%). Except for hypertension, cataract, and excessive alcohol consumption (which were excluded), there were statistically significant differences across all potential variables (see Supplementary Table 5).

#### 3.1.2 Response variable categorization

The multiple regression analysis revealed that age and education were significant variables of the Standardized Global Cognitive Score (SGCS) in the analyzed subsample (*n* = 1.185). The model explained 34.6% of the variance in SGCS (*R*^2^ = 0.346). While not exceptionally high, this indicates that education and age have a substantial impact on cognitive performance. Furthermore, the model demonstrated statistically significant explanatory power (F-statistic = 49.16, *p* < 0.000). Specifically, age was inversely correlated with SGCS (coefficient = -0.268, *p* < 0.000), whereas higher education levels were directly correlated (coefficient = 0.175, *p* < 0.000). No significant differences were found between sexes (coefficient = 0.044, *p* = 0.128).

#### 3.1.3 Data preprocessing

Missing values were present in 13 variables (see Supplementary Material 1). The proportion of missing data ranged from 10.8 to 0.1%. Since this could affect the quality of the analyses and the reliability of model results, we adopted a multiple imputation approach using the KNNImputer algorithm, beginning with the Elbow Method to determine the optimal number of neighbors. The Mean Absolute Error (MAE) ranged from 0.035 to 0.031, reflecting good overall performance of the imputation algorithm and aiding in the selection of the optimal *k-*value (see Supplementary Material 1). In addition, the data were complete for the variables sex/gender, age, educational level, social isolation, and occupational status.

#### 3.1.4 Random forest analysis

**FIGURE 2:**
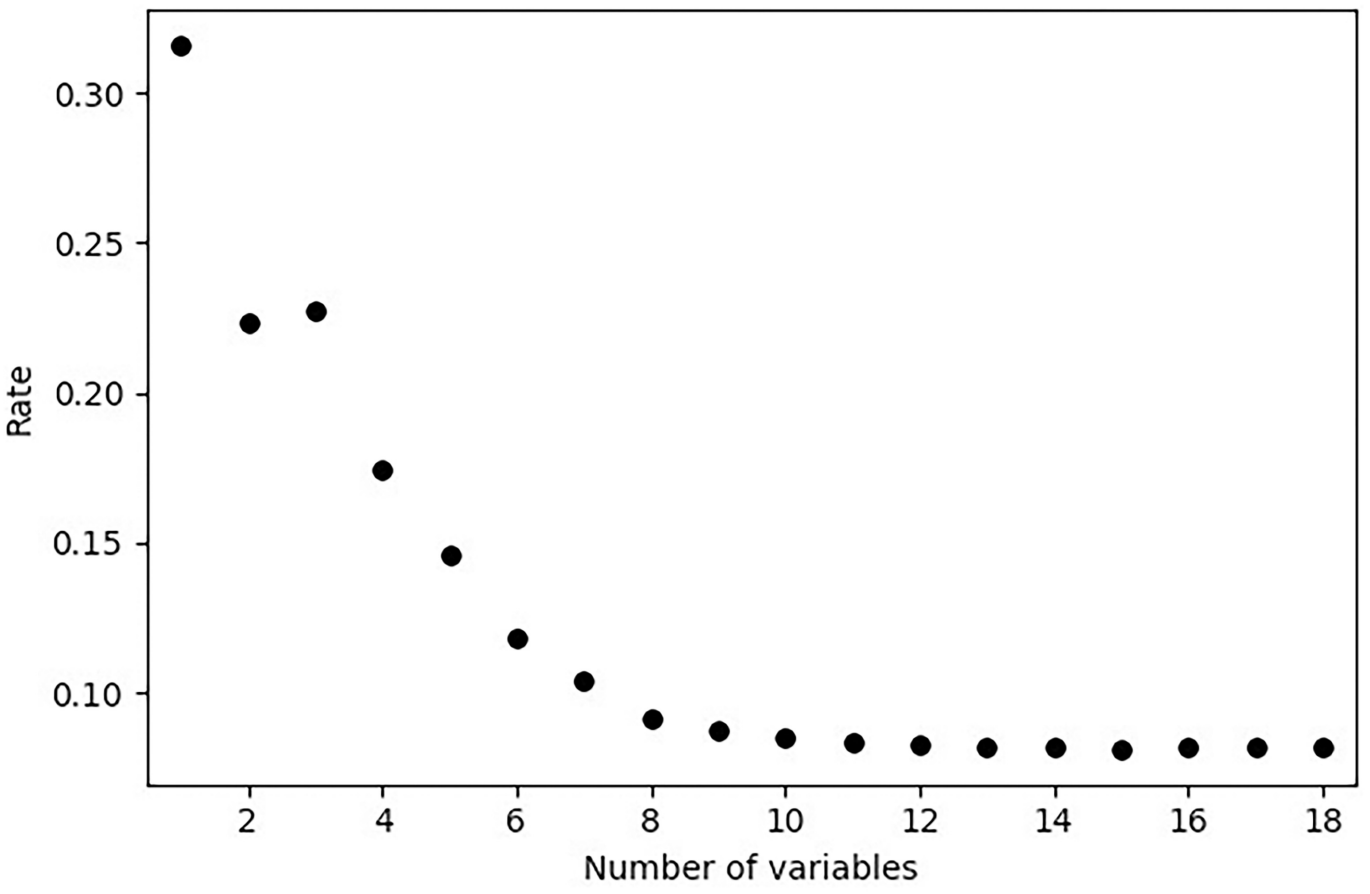
Out-of-bag error rate.

We analyzed the importance ranking of 18 variables using the RF algorithm, covering sociodemographic, clinical, lifestyle, and psychosocial data, with dementia as the dependent variable. The results showed that the top five variables, in descending order of mean decrease in impurity, were: educational level, age, life satisfaction, BMI, and HGS. The complete results can be seen in Figure 2.

**FIGURE 3:**
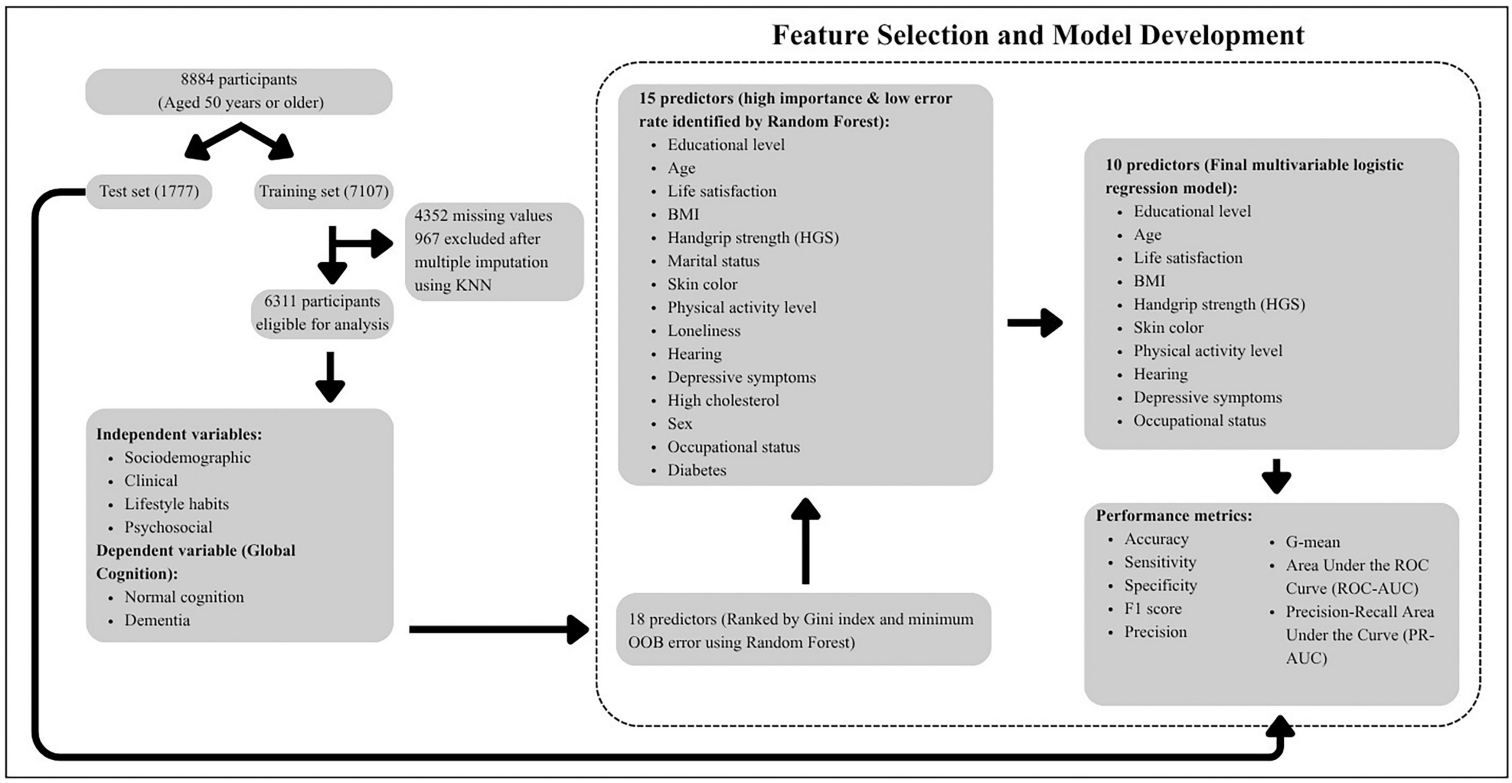
Process used for model construction.

Based on the results of the variables importance ranking, we analyzed the mean out-of-bag (OOB) error rate as variables were gradually added, in order to identify the subset that minimized error and enabled dimensionality reduction. As shown in Figure 3, the lowest OOB error rate of 0.0812% ( ± 0.0006) was achieved. All values are presented in Supplementary Table 6. This indicates that the first 16 variables, ranked by importance, yielded a low error rate in the data analysis. These variables were: educational level, age, life satisfaction, BMI, HGS, marital status, skin color, physical activity level, loneliness, hearing, depressive symptoms, high cholesterol, sex, current occupational status, and diabetes.

#### 3.1.5 Multivariable logistic regression analysis

After the initial analysis using RF, the 16 most important independent variables were selected for a multivariable LR regression analysis, with the goal of developing an interpretable model, using dementia as the dependent variable.

The variables and their respective values are presented in Supplementary Table 7. The analysis showed statistically significant results for educational level, age, life satisfaction, BMI, HGS, skin color, physical activity level, hearing, depressive symptoms, and current occupational status, all of which influenced dementia in adults aged 50 years and older (see Table 1).

**TABLE 1:** Multivariable logistic regression model analysis.

Variable	Normal cognition (*N* = 7962)	Dementia (*N* = 845)	OR (95% CI)	*P*-value
Educational level, n (%)				
Illiterate	1,031 (12.9)	332 (39.2)	7.42 (4.04–13.62)	< 0.001
Less than primary education	1,548 (19.4)	217 (25.6)	3.42 (1.88–6.22)	< 0.001 0.0343
Completed primary education	2,472 (31.0)	199 (23.5)	1.90 (1.05–3.43)	
Incomplete secondary	936 (11.7)	46 (5.4)	1.16 (0.59–2.27)	0.6609
education	1,395 (17.5)	28 (3.3)	0.44 (0.21–0.94)	0.0337
Completed secondary education	580 (7.2)	23 (2.7)	−	−
Higher education or more				
Age, n (%)				
50–54	1,927 (24.2)	134 (15.8)	−	−
55–59	1,499 (18.8)	166 (19.6)	1.62 (1.19–2.20)	0.0019
60–64	1,302 (16.3)	155 (18.3)	1.42 (1.04–1.96)	0.0298
65–69	1,028 (12.9)	144 (17.0)	1.27 (0.90–1.79)	0.1764
70–74	878 (11.0)	80 (9.4)	0.65 (0.43–1.02)	0.0406
75–79	712 (8.9)	48 (5.6)	0.28 (0.16–0.48)	< 0.001
80–84	393 (4.9)	28 (3.3)	0.15 (0.06–0.37)	< 0.001
85–89	187 (2.3)	27 (3.2)	0.28 (0.10–0.76)	0.0125
90+	36 (0.4)	63 (7.4)	11.01 (5.06–23.95)	< 0.001
Life satisfaction, median (IQR)	8.0 (5.0–10.0)	7.0 (4.0–10.0)	0.72 (0.52–0.99)	0.0462
BMI, n (%)				
Severely underweight	31 (0.4)	4 (0.5)	0.14 (0.01–1.79)	0.1316
Underweight	109 (1.4)	26 (3.4)	2.11 (1.12–3.97)	0.0210
Normal weight	2,110 (27.5)	271 (35.8)	−	−
Overweight	3,097 (40.3)	257 (33.9)	0.82 (0.64–1.03)	0.0912
Moderately obese (Grade I)	1,628 (21.2)	142 (18.7)	0.78 (0.58–1.05)	0.1011
Severely obese (Grade II)	502 (6.5)	42 (5.5)	0.76 (0.49–1.18)	0.2273
Morbid obesity (Grade III)	191 (2.4)	14 (1.8)	0.67 (0.32–1.38)	0.2736
Handgrip strength, n (%)				
High	254 (3.3)	11 (1.3)	−	−
Slightly high	823 (10.7)	46 (5.7)	1.68 (0.68–4.11)	0.2591
Moderate	1,234 (16.0)	83 (10.4)	1.62 (0.68–3.85)	0.2750
Slightly low	2,217 (28.8)	171 (21.4)	1.74 (0.75–4.03)	0.1995
Low	3,162 (41.1)	485 (60.9)	2.50 (1.09–5.76)	0.0313
Marital status, n (%)				
Single	848 (10.6)	112 (13.2)	1.00 (0.72–1.39)	0.9771
Married/partner/stable union	4,705 (59.0)	410 (48.5)	−	−
Divorced or separated	978 (12.2)	98 (11.6)	0.77 (0.56–1.06)	0.1147
Widowed	1,431 (17.9)	225 (26.6)	1.11 (0.83–1.48)	0.5003
Skin color, n (%)				
White	3,157 (40.9)	248 (31.3)	−	−
Black	713 (9.2)	114 (14.4)	1.47 (1.07–2.00)	0.0157
Educational level, n (%)				
Skin color, n (%)				
Brown (mixed race)	3,589 (46.5)	395 (50.0)	1.04 (0.83–1.31)	0.7306
Yellow (East Asian origin)	73 (0.9)	9 (1.1)	1.26 (0.51–3.09)	0.6198
Indigenous	183 (2.3)	24 (3.0)	1.01 (0.52–1.97)	0.9660
Physical activity level, n (%)				
High	2,330 (30.9)	149 (19.3)	−	−
Moderate	2,190 (29.1)	155 (20.1)	1.14 (0.87–1.50)	0.3501
Low	3,004 (39.9)	467 (60.5)	1.61 (1.25–2.08)	< 0.001
Loneliness, n (%)				
Never	3,765 (52.0)	276 (42.8)	−	−
Sometimes	2,349 (32.4)	165 (25.6)	0.93 (0.72–1.19)	0.5538
Always	1,124 (15.5)	203 (31.5)	1.23 (0.93–1.64)	0.1529
Hearing, n (%)				
Good	5,554 (69.8)	525 (62.4)	−	−
Fair	1,985 (24.9)	197 (23.4)	0.93 (0.74–1.18)	0.5675
Poor	416 (5.2)	119 (14.1)	1.65 (1.16–2.37)	0.0059
Depressive symptoms, n (%)				
No	4,904 (66.8)	302 (45.9)	−	−
Yes	2,434 (33.1)	355 (54.0)	1.72 (1.36–2.16)	< 0.001
Sex, n (%)				
Male	3,522 (44.2)	312 (37.0)	−	−
Female	4,440 (55.7)	532 (62.9)	1.07 (0.86–1.34)	0.5531
High cholesterol, n (%)				
No	5,453 (69.0)	554 (66.6)	−	−
Yes	2,447 (30.9)	277 (33.3)	1.12 (0.91–1.39)	0.2805
Occupational status, n(%)				
No (not working)	5,465 (68.6)	691 (81.7)	−	−
Yes (working)	2,497 (31.3)	154 (18.2)	0.78 (0.61–1.00)	0.0463
Diabetes, n (%)				
No	6,669 (84.1)	666 (79.5)	−	−
Yes	1,259 (15.8)	171 (20.4)	1.02 (0.78–1.33)	0.8986

BMI, Body Mass Index; IQR, Interquartile Range.

#### 3.1.6 Model performance

Using the test set, final models were evaluated based on the 10 independent variables that were significantly associated with dementia. The RF model achieved an Area Under the ROC Curve (AUC-ROC) of 0.776 (95% CI: 0.740–0.811), with sensitivity (recall) of 0.708, specificity of 0.702, precision of 0.201, Precision-Recall Area Under the Curve (PR-AUC) of 0.261 (95% CI: 0.217–0.319), F1 score of 0.311, G-means of 0.705, and accuracy of 0.703.

The LR model showed a similar but slightly lower performance, with an AUC-ROC of 0.735 (95% CI: 0.697–0.773), sensitivity (recall) of 0.678, specificity of 0.654, precision of 0.171, PR-AUC of 0.221 (95% CI: 0.183–0.275), F1 score of 0.272, G-means of 0.666, and accuracy of 0.656.

### 3.2 Discussion

In this study, we used data collected from the Brazilian Longitudinal Study of Aging (ELSI-Brazil), one of the largest population-based studies in Brazil, with national representation of non-institutionalized individuals aged 50 years and older. Our objective was to develop a simple classification model to identify probable dementia based on sociodemographic and health-related variables in middle-aged and older Brazilian adults, by analyzing sociodemographic, health, lifestyle, and psychosocial factors and identifying which of them were most relevant in building the model.

General characteristics of the sample are presented in Supplementary Table 5. Initially, the study included 9,412 participants. After applying the neuropsychological assessments and informant-based cognitive function evaluation, we were able to classify individuals into three cognitive status groups: normal cognition, cognitive impairment, and dementia. For the purposes of the present analysis, we excluded participants classified as having cognitive impairment without dementia (*n* = 528) in order to focus on the distinction between cognitively normal individuals and those with dementia. The final analytical sample therefore included 8,884 participants.

#### 3.2.1 Prevalence of dementia among middle-aged and older adults in Brazil

The results of this study revealed that the prevalence of dementia among Brazilian adults aged 50 and older was 9.6%, a value higher than that reported in a recently published study, which estimated the prevalence of all-cause dementia in Brazil at 8.5% (Aliberti et al., 2025). However, it is important to note that the authors of that study included only individuals aged 60 and over, whereas the present study assessed cognition beginning at age 50. Although dementia traditionally affects older individuals, there is growing evidence that it may manifest—albeit in milder forms—at increasingly earlier ages (Jaakkimainen et al., 2022; Loi et al., 2023; Yuan et al., 2021).

A general trend of increasing dementia prevalence with advancing age was observed. The lowest prevalence occurred among participants aged 50–54 years (6.5%), while the highest was found in the 90+ age group, reaching 62.4%. This finding is consistent with well-established knowledge that dementia predominantly affects individuals in older age groups (Cao et al., 2020).

As in the study by Aliberti et al. (2025), which reported higher prevalence among women (9.1%) compared to men (7.7%), our results also reflect this trend, with rates of 10.7% for women and 8.2% for men. These findings align with multiple studies suggesting that women face an increased lifetime having dementia, potentially due to biological, social, and cognitive factors (GBD 2019 Dementia Forecasting Collaborators, 2022; Ibeachu, 2023; Liu et al., 2023; Reisz et al., 2021).

We also analyzed dementia prevalence by educational level and found that it was highest among illiterate participants (24.4%), approximately two to three times higher than in intermediate education levels with 12.3% for those with less than primary education and 7.5% for those who completed primary school. Prevalence was 4.7% among those with incomplete secondary education and lowest among those with complete secondary education (2.0%). A slight increase was observed in the group with higher education or more (3.8%). This inverse pattern—lower prevalence with higher educational attainment—reinforces findings from previous studies in Brazil and elsewhere, in which education is shown to serve as a marker of cognitive reserve and a protective factor against dementia onset (Gonçalves et al., 2023; Lopes et al., 2024).

#### 3.2.2 Random forest algorithm and multivariable logistic regression analysis of dementia among middle-aged and older adults in Brazil

The RF algorithm and a multivariable LR analysis were conducted in this study to investigate factors influencing dementia in Brazilian adults aged 50 years and older. After adjustments, the results revealed that the odds of dementia were 7.42 times higher (OR = 7.42; 95% CI: 4.04–13.62; *p* < 0.001) for illiterate individuals. As educational level increased, the odds decreased, though they remained elevated for those with low education. Individuals with less than primary education had 3.42 times greater odds (OR = 3.42; 95% CI: 1.88–6.22; *p* < 0.001), and those who completed primary education had 90% higher odds (OR = 1.90; 95% CI: 1.05–3.43; *p* = 0.034). In contrast, those who completed high school showed a 56% reduction in the odds (OR = 0.44; 95% CI: 0.21–0.94; *p* = 0.033) compared to individuals with a university degree or higher. The group with incomplete high school, although showing lower odds (OR = 1.16), did not exhibit a statistically significant association (95% CI: 0.59–2.27; *p* = 0.660).

Educational level has been consistently included as a variable in classification models developed in population-based studies due to their availability and consistent contribution to model performance (Hou et al., 2019). Education attainment can function as a stable proxy variable that helps improve discrimination between individuals with and without dementia in population-based models. In the Brazilian context, where educational inequalities remain substantial across older generations (Esteves, 2024), this variable may be particularly informative for classification models that rely on easily obtainable information. However, the findings in this study may reflect not only the absence of formal education—as typically categorized in epidemiological studies—but also broader structural inequalities, such as poverty, limited access to healthcare, less cognitively demanding occupations, and reduced engagement in cognitively stimulating activities throughout life, representing a trajectory of cumulative social disadvantage (Maccora et al., 2020).

The relationship between age and dementia is often complex, influenced by various interacting factors. However, dementia prevalence generally increases with advancing age across all causes (Cao et al., 2020). In our findings, individuals aged 90 and above exhibited significantly higher odds of dementia compared to the youngest age group (OR = 11.01; 95% CI: 5.06–23.95; *p* < 0.001), which aligns with well-established literature (Cao et al., 2020). Interestingly, this trend was not observed in the age groups between 70 and 89 years. In fact, the odds ratios indicated lower chances of dementia in individuals aged 70–74 years (OR = 0.65; 95% CI: 0.43–1.02; *p* = 0.0406), although the confidence interval includes an OR of 1.00 (suggesting equal odds between groups). The odds were also significantly lower for those aged 75–79 years (OR = 0.28; 95% CI: 0.16–0.48; *p* < 0.001), 80–84 years (OR = 0.15; 95% CI: 0.06–0.37; *p* < 0.001), and 85–89 years (OR = 0.28; 95% CI: 0.10–0.76; *p* = 0.0125).

This seemingly paradoxical association may be explained through the lens of survivor bias. According to mortality data from DATASUS for the years 2015–2016, all-cause mortality increased from 58.5 per 100,000 inhabitants in the 70–74 age group to 182.5 in individuals aged 80 and over (Informações de Saúde (TABNET) – Datasus, 2024). Survivor bias arises when individuals who live to older ages and remain cognitively intact represent a more resilient subgroup, while more vulnerable individuals may have developed dementia-related pathologies earlier and died before reaching older ages, thus being excluded from the observed sample. As a result, dementia cases may be underrepresented in older age groups within cross-sectional samples, potentially leading to an underestimation of the true odds of dementia in these age brackets (Elston, 2021). Statistically, high mortality functions as a selective filter in observational data—reducing the denominator in LR models—which can lead to artificially lower odds ratios, creating the illusion of a protective effect of advanced age. Despite this possible explanation, these findings should be interpreted with caution. The cross-sectional nature of this study limits causal inference and introduces the potential for bias. Longitudinal studies are needed to better elucidate age-related cognitive trajectories and to validate these findings.

Our findings indicate that engaging in any occupational activity, such as paid work in the past 30 days, was associated with a 22% reduction in the odds of dementia compared to not performing occupational activities (OR = 0.78; 95% CI: 0.61–1.00; *p* = 0.0463) with the best-case scenario (based on the lower confidence limit) indicating up to 39% lower odds. These findings reinforce the relevance of social and life context variables and suggest that occupational activity contributes to distinguishing individuals with dementia from cognitively normal participants in the model. However, the confidence interval approaches the null value, and the result lies at the threshold of statistical significance, indicating that the magnitude of the association may be modest. Occupational engagement has been previously reported in epidemiological studies of cognitive aging, where employment trajectories across adulthood have been associated with differences in later-life cognitive performance (Pacca et al., 2025). Moreover, work promotes autonomy and fosters long-term social exposure, which directly impacts social networks (Kivimäki et al., 2021; Pacca et al., 2025). It is important to note that the indicator used in this study captures only whether an individual is currently engaged in occupational activity, it does not assess the type of job, the complexity of tasks, or the duration of occupational exposure, which are key factors that influence cognitive demands. Future studies using more detailed occupational measures may further clarify the role of work-related characteristics in dementia classification models.

In addition to maintaining occupational engagement throughout life, another key social marker of vulnerability, skin color, emerges as a relevant factor in our analysis, revealing a structural inequality that directly impacts brain and cognitive health. Our findings indicate that self-identified black individuals had significantly higher odds of dementia compared to self-identified white individuals (OR = 1.47; 95% CI: 1.07–2.00; *p* = 0.0157). This result aligns with previous Brazilian studies highlighting ethnic-racial disparities in dementia burden, including higher dementia-related mortality rates among black populations (Rosa et al., 2024).

Interestingly, cognitive and functional abilities appear to be similar between black and white individuals, and race has not been shown to modify the associations between cognition and neuropathology (Suemoto et al., 2024). Therefore, our results are likely to race as a marker of socio-structural inequality. In the Brazilian context, self-reported skin color should be interpreted primarily as a social and structural marker rather than a biological determinant of disease. In Brazil, black populations continue to face perceived barriers in accessing healthcare services, lower educational attainment with higher dropout rates in basic education, and limited access to more qualified employment opportunities (Anunciação et al., 2022; Constante et al., 2021), even when educational credentials are comparable to those of white individuals (Rodrigues, 2021). In a highly diverse and racially mixed population, differences in health outcomes often reflect these inequalities.

These findings highlight the presence of social and structural vulnerabilities, notably low educational attainment, economic instability, and social insecurity, all of which are social determinants that negatively affect healthy brain aging in the Brazilian population (Da Ros et al., 2025). Including this variable in the model therefore allows the identification of potential structural determinants associated with dementia. However, the use of sensitive attributes in machine learning requires careful ethical consideration, as classification model may inadvertently reproduce existing health disparities. As highlighted by Chen et al. (2021), responsible machine learning in healthcare should consider fairness, transparency, and the broader social context in which models are developed and applied.

Aging is characterized by a series of changes resulting from cumulative psychosocial, physiological, and neurological processes throughout life, which can negatively impact cognitive health. Among these changes, sensory loss, such as reduced hearing acuity, has gained increasing attention as one of the most relevant modifiable risk factors for dementia prevention (Avan and Hachinski, 2022). Self-perceived hearing ability is a critical indicator of cognitive health. In our analysis, participants who reported regular hearing showed no statistically significant association with dementia (OR = 0.93; 95% CI: 0.74–1.18; *p* = 0.5675). However, individuals who reported poor hearing had 65% higher odds of dementia compared to those who perceived their hearing as good (OR = 1.65; 95% CI: 1.16–2.37; *p* = 0.0059).

Previous epidemiological studies (Paiva et al., 2023; Samelli et al., 2025) have reported similar associations between hearing impairment and cognitive decline, suggesting that sensory function may be an important marker of cognitive health in aging populations. Moreover, it is estimated that up to 6.8% of dementia cases could potentially be prevented in the general population if hearing loss were eliminated (Suemoto et al., 2023). From a modeling perspective, the inclusion of self-reported hearing status may contribute to improving classification performance using variables that are easily obtainable in primary care settings. These findings reinforce the potential value of simple sensory assessments as part of screening-oriented approaches for identifying individuals who may benefit from further cognitive evaluation, aiming to enhance brain health in the Brazilian population.

In this context of modifiable physiological factors related to brain and cognitive health, nutritional status—as reflected by BMI—emerges as a relevant variable in the classification model. The only statistically significant association was observed among individuals classified as underweight (BMI: 16.0–18.4 kg/m^2^), who had more than twice the odds of dementia compared to those with normal weight (OR = 2.11; 95% CI: 1.12–3.97; *p* = 0.0210). Although overweight and obesity are currently in the spotlight as major risk factors for dementia (Livingston et al., 2024), previous epidemiological studies have also reported associations between low body weight and dementia, suggesting that underweight status may function as a potential marker of preclinical dementia. Some studies argue that weight loss may begin years prior to clinical diagnosis, possibly reflecting metabolic or behavioral changes as subclinical expressions of prodromal dementia (Lee et al., 2020). The inclusion of BMI may therefore contribute to improving the classification of individuals with probable dementia using simple health indicators that are routinely collected and easily integrated into primary care assessments. Therefore, while our findings highlight the potential relevance of nutritional status as a marker of cognitive vulnerability in aging populations, the nature of this relationship remains uncertain. Further research is needed to clarify this link in adults aged 50 years and older.

Just as nutrition plays a key role, various lifestyle factors with modifiable potential and direct influence on brain health have gained increasing attention (Livingston et al., 2024), among which physical activity stands out as one of the most consistent pillars in promoting cognitive and brain health (Jiang et al., 2025; Kong et al., 2025). Our findings demonstrated that individuals with lower levels of physical activity had a 61% higher chance of dementia compared to those with high levels of activity (OR = 1.61; 95% CI: 1.25–2.08; *p* < 0.001). This finding aligns with epidemiological evidence suggesting that regular engagement in, or higher levels of, physical activity has protective effects on outcomes related to brain and cognitive health (Cunningham et al., 2020). In the present study, physical activity level—assessed using the IPAQ—contributed meaningfully to the performance of the classification model. Therefore, behavioral indicators such as physical activity level may provide valuable information for identifying individuals with greater cognitive vulnerability using measures that can be easily obtained in primary care settings. From a public health perspective, the encouraging and sustaining regular physical activity among middle-aged and older adults in Brazil should be recognized as a public health priority, aimed at strengthening brain health, maintaining cognitive function, and supporting healthy aging.

Within this same framework of physical functionality as a favorable factor for brain health, HGS stands out as an objective marker for assessing muscular strength an element directly associated with functional and cognitive decline in middle-aged and older adults (Xie et al., 2025). Although the intermediate HGS categories (slightly high, moderate, and slightly low) were not statistically significant in our study, we found a significant association between low HGS (see Supplementary Table 4 for reference values). Specifically, individuals with low HGS had 2.5 times higher odds of dementia compared to those with high HGS (OR = 2.50; 95% CI: 1.09–5.76; *p* = 0.0313). The multicenter study coordinated by Zammit et al. (2019) revealed a significant correlation between HGS decline and impairments across multiple cognitive domains. Thus, HGS serves as a low-cost functional marker that reflects the integrity of the central nervous system, brain plasticity, functional reserve, and resilience, all essential elements for healthy brain aging (Zammit et al., 2019). Systematic screening of HGS in middle-aged and older adults, followed by exercise-based interventions aimed at improving muscle strength and reducing functional limitations, may represent a key strategy for the prevention and delay of dementia in the Brazilian population. Such actions could contribute not only to the preservation of functional capacity but also to the promotion of healthy brain aging.

In addition to physical and functional aspects, psychosocial factors are frequently examined in relation to cognitive health in aging populations, with depressive symptoms and cognitive decline in middle-aged and older adults being consistently investigated worldwide (Boparai et al., 2025; Li et al., 2025). Our findings revealed that individuals exhibiting depressive symptoms had 72% greater odds of dementia (OR = 1.72; 95% CI: 1.36–2.16; *p* < 0.001). This result is strongly aligned with a recent meta-analysis, which found that older adults with depression were 1.75 times more likely to develop dementia (da Costa Galvão et al., 2025). Depressive symptoms emerged as an important psychosocial indicator in distinguishing individuals with different cognitive profiles. This variable may capture broader behavioral and psychosocial patterns frequently associated with cognitive vulnerability, including lower levels of physical activity, social disengagement, and reduced adherence to healthy lifestyles.

Continuing the analysis of psychosocial factors, subjective wellbeing perception stands out as an important marker of cognitive health. In our findings, life satisfaction emerged as a key psychological indicator, reflecting how content individuals feel with their lives overall or in specific domains. Among middle-aged and older adults, each unit increase in life satisfaction was associated with 28% lower odds of dementia (OR = 0.72; 95% CI: 0.52–0.99; *p* = 0.0462). This suggests that a positive self-perception of life may serve as a protective factor against cognitive decline. Evidence shows that higher levels of eudaimonic wellbeing—defined by a sense of purpose, personal growth, self-acceptance, and positive relationships—have been strongly linked to better cognitive functioning and cognitive resilience, the brain’s capacity to adapt to stress and recover from mental and emotional adversity (Willroth et al., 2023). Furthermore, family bonds and personal development may play important roles as sources of life satisfaction and perceived life meaning (Dewitte et al., 2021). Individuals with higher satisfaction levels are more likely to engage in cognitively stimulating routines, maintain social interactions, and adopt healthy lifestyle habits—factors that contribute to brain health and protect cognition throughout the aging process.

Interpreting our findings also requires considering differences between HICs and LMICs in dementia risk profiles, diagnostic access, and health-system capacity. Evidence from epidemiological studies indicates that modifiable risk factors play a substantial role in dementia globally; however, their prevalence and population impact appear to be higher in LMIC settings. In Brazil, for example, recent estimates suggest that 59.5% of dementia cases may be attributable to modifiable factors such as low educational attainment, untreated visual impairment, and depression, highlighting the particularly large potential for prevention in middle-income contexts (Suemoto et al., 2025).

In addition to differences in exposure to risk factors, structural disparities in health systems influence the detection and management of dementia. Studies comparing Brazil and the United Kingdom show that dementia remains substantially underdiagnosed in Brazil, with a large proportion of cases not formally identified due to limited availability of specialists, restricted access to diagnostic resources, and socioeconomic barriers that affect healthcare utilization (Calil et al., 2020). At the same time, cross-national analyses indicate that although countries with more developed health systems have greater diagnostic infrastructure and established care pathways, they still face capacity constraints and growing demand associated with population aging (Durgante et al., 2020).

In Brazil specifically, limitations in specialist availability and health-system capacity may delay diagnostic evaluation and access to emerging therapies, reflecting broader structural challenges in LMIC health systems (Mattke et al., 2023). It’s estimates that the waiting list for specialist consultations will increase from approximately 400,000 in 2022 to over 2.2 million by 2040, due to the limited availability of dementia specialists. Brazil currently has only 2.7 specialists per 100,000 inhabitants, compared to an average of 11.27 in G7 countries. This shortage may result in prolonged waiting times for treatment, potentially reaching up to 2 years on average in the public healthcare sector (Mattke et al., 2023). For this reason, scalable technologies and preventive strategies are essential to help mitigate the challenges faced by the Brazilian healthcare system.

Despite these challenges, the rapid expansion of dementia research initiatives and epidemiological cohorts in Brazil provides an important opportunity to generate locally relevant evidence and develop prevention strategies tailored to LMIC populations (Lourenco, 2026). Taken together, these contextual differences underscore the importance of developing predictive models and public health strategies that are adapted to the realities of LMIC settings, where scalable and low-cost approaches for early identification of individuals at higher risk of dementia may have substantial impact on disease prevention and health-system planning.

#### 3.2.3 Potential applicability in primary health care

These findings enable regular monitoring of potential factors that may contribute to cognitive decline and play a crucial role in encouraging lifestyle changes and, in some cases, early treatment adherence. Such measures can help reduce the burden of cognitive decline and, consequently, dementia in the Brazilian population. By integrating the identification of vulnerable populations and the assessment of these factors into primary healthcare, it is possible to develop effective and accessible prevention strategies, yielding benefits both at the individual level and for the public health system.

Dementia is one of the leading causes of cognitive impairment and disability among the older population and has been steadily increasing due to population aging and modifiable risk factors that contribute to higher prevalence rates (Borelli et al., 2022a; Lazzaretti et al., 2025; Livingston et al., 2024). This trend is particularly exacerbated in LMICs such as Brazil, compared to high-income countries (Borelli et al., 2022b). The situation in Brazil is especially concerning, with an estimated 80% of dementia cases among individuals aged 60 and older going undiagnosed—particularly in poorer regions and among the younger old (ages 60–64—making timely intervention and management more difficult. This scenario highlights a critical public health challenge (Miguel et al., 2024a; Miguel et al., 2024b).

Given this context, early detection can significantly improve individual patient management and support more efficient allocation of healthcare resources. Currently, diagnosing dementia is costly and time-consuming, requiring detailed clinical history, physical examination, cognitive assessments, and additional imaging and laboratory tests. With the aging population, the number of new dementia cases is expected to rise, placing even greater strain on healthcare systems. Since a large portion of cases remain undiagnosed, the risk of complications due to inadequate management increases.

Classification model using machine learning for the detection of cognitive impairment and/or dementia are increasingly being explored worldwide, including growing efforts in LMICs (Brain et al., 2024; Javeed et al., 2023). However, many of these models rely on high-cost data sources such as imaging, clinical biomarkers, voice analysis, or genetic data, factors that limit their practical implementation in primary care settings (Javeed et al., 2023). An alternative approach, however, involves the use of easily obtainable and low-cost variables, as demonstrated in models developed by Ren et al. (2022) using UK data, and Wang et al. (2025) using data from China.

Systematic reviews have highlighted a clear gap in the use of clinically accessible variables (Javeed et al., 2023; Kumar et al., 2021). This underscores the importance of developing models based on practical, easy-to-assess variables that can be collected during routine primary care visits in Brazil using simple exams and standardized questionnaires. A notable example is the AGES-Reykjavik study by Twait et al. (2023), which utilized a range of accessible clinical variables to predict dementia in the general population. In their study, the RF algorithm achieved an AUC-ROC of 0.71 (95% CI: 0.68–0.74), with 55% sensitivity and 75% specificity. These results were comparable to those from traditional statistical methods such as LR (AUC-ROC: 0.71; 95% CI: 0.68–0.74), with 64% sensitivity and 68% specificity.

Similarly, our findings showed comparable performance, with the RF model outperforming LR across nearly all metrics. It demonstrated higher discriminative ability (AUC-ROC: 0.776; 95% CI: 0.740–0.811 vs. 0.735; 95% CI: 0.697–0.773), better balance between identifying and excluding cases (sensitivity 70.8% and specificity 70.2% vs. 67.8 and 65.4%), a superior G-mean (0.705 vs. 0.666), and higher overall accuracy (0.703 vs. 0.656). In both models, however, the F1 score remained low (0.311 for RF and 0.272 for LR), indicating modest performance in detecting the positive class and a relatively high rate of false positives.

The RF model demonstrated a moderate yet meaningful discriminative capacity. The ROC-AUC of 0.776 (95% CI: 0.740–0.811) indicates that the model is able to distinguish between individuals with and without the outcome substantially better than chance. Importantly, the model achieved a balanced performance between sensitivity (recall) (0.708) and specificity (0.702), reflected by a G-mean of 0.705, suggesting that it can reasonably identify both positive and negative cases. From a practical perspective, this balance is valuable in population-level screening contexts, where missing true cases may have important consequences. However, the relatively low precision (0.201) and modest PR-AUC (0.261; 95% CI: 0.217–0.319) indicate that a considerable proportion of predicted positive cases correspond to false positives, which is expected in the presence of substantial class imbalance and low outcome prevalence. Consequently, while the model shows promise as a screening support tool, it is predictions should be interpreted cautiously and ideally used in combination with additional clinical or epidemiological information. In practical settings, such models may be more appropriate for identifying individuals who could benefit from further assessment rather than serving as a definitive diagnostic tool. Future work should explore strategies to improve precision, including alternative modeling approaches, recalibration of decision thresholds, or integration of additional predictive features.

This study contributes to advancing research in this field by using nationally representative data of Brazilian adults aged 50 and older. It combines the RF algorithm, which handles complex interactions and nonlinearities even in the presence of collinearity, with LR, which allows for clearer interpretation through odds ratio estimates. This hybrid approach enabled the identification of low-cost variables—sociodemographic, clinical, lifestyle, and psychosocial factors—that are easily assessable in primary care settings and useful for classifying cognitive status in middle-aged and older adults.

These findings have important public health implications, particularly for primary care, where early identification of potential neurodegenerative disease can trigger further investigation and health education. Moreover, they support the implementation of early lifestyle interventions, which have shown significant potential in improving cognitive function in individuals with early cognitive impairment or Alzheimer’s disease (Ornish et al., 2024), ultimately promoting better quality of life and longevity.

#### 3.2.4 Limitations and implications

There are several limitations to our study. The cross-sectional nature of the dataset prevents the establishment of causal relationships between variables and outcomes. As such, the results reflect associations only and do not allow for determination of causality. Furthermore, because dementia is a progressive condition, the data used in this study cannot capture temporal dynamics, leading to a loss of information about disease progression or interactions between predictors over time. This limits the generalizability of findings to other time points, particularly in changing contexts. Future research could benefit from using longitudinal data from ELSI-Brazil, allowing for a more robust analysis of the relationships between predictors and outcomes, as well as the development of predictive models of cognitive status.

An important limitation is related to the use of self-reported variables included in the model, such as diabetes, high cholesterol, hearing loss, alcohol consumption, and cataract status. Self-reported measures are subject to recall bias, misclassification, and potential underreporting, particularly among older adults. For instance, individuals may be unaware of certain clinical conditions or may inaccurately report lifestyle behaviors. Such measurement errors may lead to misclassification of variables, potentially attenuating associations or reducing the performance of the model. In the context of machine learning, misclassification in variables can introduce noise into the training data, which may partially explain the moderate performance observed. Future studies incorporating clinically verified diagnoses or objective measurements may improve the precision of the variables, model robustness and predictive accuracy.

Another limitation concerns the absence of external validation. The model was developed and internally evaluated using the same dataset, which may limit it’s generalizability to other populations. Although internal validation provides an initial assessment of model performance, it does not guarantee that similar accuracy would be observed in independent samples or different clinical settings. Population characteristics, measurement procedures, and prevalence of dementia may vary across cohorts, potentially affecting model performance. Therefore, the proposed model should be interpreted as a preliminary classification framework. Future studies should evaluate it is robustness through external validation using independent datasets or longitudinal cohorts.

This study also has limitations related to the ethical implications of including sensitive attributes such as skin color in classification models. Although this variable was included to better understand structural determinants of health in the Brazilian context, we did not perform subgroup fairness analyses (e.g., evaluating differences in sensitivity, specificity, or calibration across population groups). Future studies should investigate the potential for differential model performance and assess fairness metrics to ensure that tools do not inadvertently reinforce existing health disparities.

Regarding the exclusion of participants with cognitive impairment without dementia from analysis. Although this strategy allowed a clearer binary distinction between normal cognition and dementia, it may simplify the complexity of the cognitive aging continuum. Future studies could explore multinomial predictive models that incorporate cognitive impairment as an intermediate category, potentially providing a more comprehensive representation of cognitive trajectories in aging populations.

Another methodological consideration concerns the choice of a categorical outcome rather than a continuous probability score of dementia. In the present study, our objective was to develop a simple screening-oriented model that could be applied in primary care settings using easily obtainable variables. In clinical practice, screening tools are typically designed to classify individuals who may require further diagnostic evaluation rather than to estimate continuous probabilities of disease. Therefore, a categorical classification approach was considered more appropriate for the practical application of the proposed model.

Finally, Brazil presents substantial regional socioeconomic disparities that may influence dementia risk profiles, access to diagnosis, and health outcomes. Wealthier regions such as the South and Southeast generally have greater availability of specialized healthcare services and higher socioeconomic indicators compared with the North and Northeast regions. These structural differences may affect both the distribution of modifiable risk factors and the probability of dementia detection. Although the present study used nationally representative data, the analysis was not designed to investigate potential regional heterogeneity in model performance or associations between predictors and dementia. Future studies should explore whether predictive models perform differently across regions with distinct socioeconomic characteristics, potentially incorporating indicators such as regional GDP per capita or household income data to better understand regional disparities in dementia risk and detection.

The strengths of this study include the large sample size, the wide range of collected variables, and the diversity of the population, encompassing individuals of different skin colors, as well as it’s representativeness of adults aged 50 and older in Brazil. The ELSI-Brazil study incorporated a comprehensive battery of neuropsychological tests designed to provide a broad cognitive assessment. This allowed for the calculation of global cognitive scores using regression-based norms that accounted for the effects of education and age. In contrast, other studies predicting cognitive impairment and dementia have often relied solely on brief screening tools such as the Mini-Mental State Examination (Na, 2019; Nyholm et al., 2024; Twait et al., 2023). Moreover, ELSI-Brazil included an informant-based cognitive function section, which enabled the assessment of individuals unable to complete the neuropsychological tests, thereby complementing the evaluation and minimizing sample loss.

## 4 Conclusion

According to the results of this study, we observed a prevalence of dementia among Brazilian adults aged 50 years and older that is higher than previously reported in earlier studies. By combining two analytical approaches—RF and multivariable LR—we developed a practical model to classify probable dementia using 10 low-cost and interpretable variables derived from sociodemographic and health-related variables. The analyses revealed that low educational attainment, older age, low body mass index, reduced handgrip strength, self-reported Black race, physical inactivity, self-reported hearing loss, and the presence of depressive symptoms were consistently associated with higher odds of dementia. Conversely, higher educational attainment, better self-perceived life satisfaction, and currently being employed emerged as protective factors. These variables encompass sociodemographic, clinical, lifestyle, and psychosocial factors. Their inclusion in the model underscores their relevance in understanding the health determinants related to cognitive status. Moreover, they are accessible within primary care contexts and have the potential to be used in identifying individuals at cognitive and brain vulnerability, especially as such conditions are becoming a leading concern in LMICs, particularly in Brazil, where the health system faces challenges such as inadequate infrastructure, a shortage of dementia specialists, and inequitable access to care. It is therefore essential to focus on integrated approaches that improve early screening in primary care by identifying clinically accessible and relevant factors for preventive strategies. This can enhance resource allocation, both economic and human, while improving the accuracy of timely interventions aimed at preventing or delaying the onset of dementia. Such measures may contribute to addressing the growing burden of dementia in Brazil. Given the cross-sectional nature of this study, we recommend that future research employ longitudinal data and independent cohorts to deepen the temporal understanding of the relationship between variables and dementia, and to validate the findings observed.

## Data Availability

The data used in this study come from the Longitudinal Study of the Health of Brazilian Elderly (ELSI-Brasil), a database under restricted access and institutional control, whose availability depends on the formal submission of a project to the team responsible for data collection, followed by ethical analysis and specific approval. We do not have the rights to publicly share the data, and we do not have authorization for independent redistribution of the dataset used. Therefore, it is not possible to make the data available. It is possible to establish contact or to request access directly from the database management team through the website^1^ by submitting a proposal according to the guidelines established by the responsible institution. We remain available to provide additional information about the access request process, if necessary.

## Supplementary material

Supplementary Material 1 Missing data and elbow method.

Supplementary Table 1 Neuropsychological test battery.

Supplementary Table 2 Informant Questionnaire on Cognitive Decline in Older Adults (IQCODE), short version with 16 items.

Supplementary Table 3 Variables used for subsample selection.

Supplementary Table 4 Absolute hand grip strength.

Supplementary Table 5 Comparison of the occurrence of cognitive dysfunction between predictors.

Supplementary Table 6 Random forest analysis using the out-of-bag (OOB) error curve.

Supplementary Table 7 Variables and their respective values.
