# Supplementary material for: Construction of a classification model for dementia among Brazilian adults aged 50 and over
Source: Front Aging Neurosci. 2026 Apr 15;18:1789012. doi: 10.3389/fnagi.2026.1789012 (PMC13126550; doi:10.3389/fnagi.2026.1789012)
Supplement: Supplementary Material 1 — Missing data and elbow method. [file Data_Sheet_1.docx]

Amount of Missing Data and Elbow Method

Analysis of missing data in the dataset. Description of the variables: 1 = Loneliness; 2 = Retinopathy; 3 = Life Satisfaction; 4 = Depressive Symptoms; 5 = Level of Physical Activity; 6 = BMI; 7 = FPM; 8 = Skin Color; 9 = Cholesterol; 10 = Cognitive Status (target); 11 = Diabetes; 12 = Smoking; 13 = Hearing.
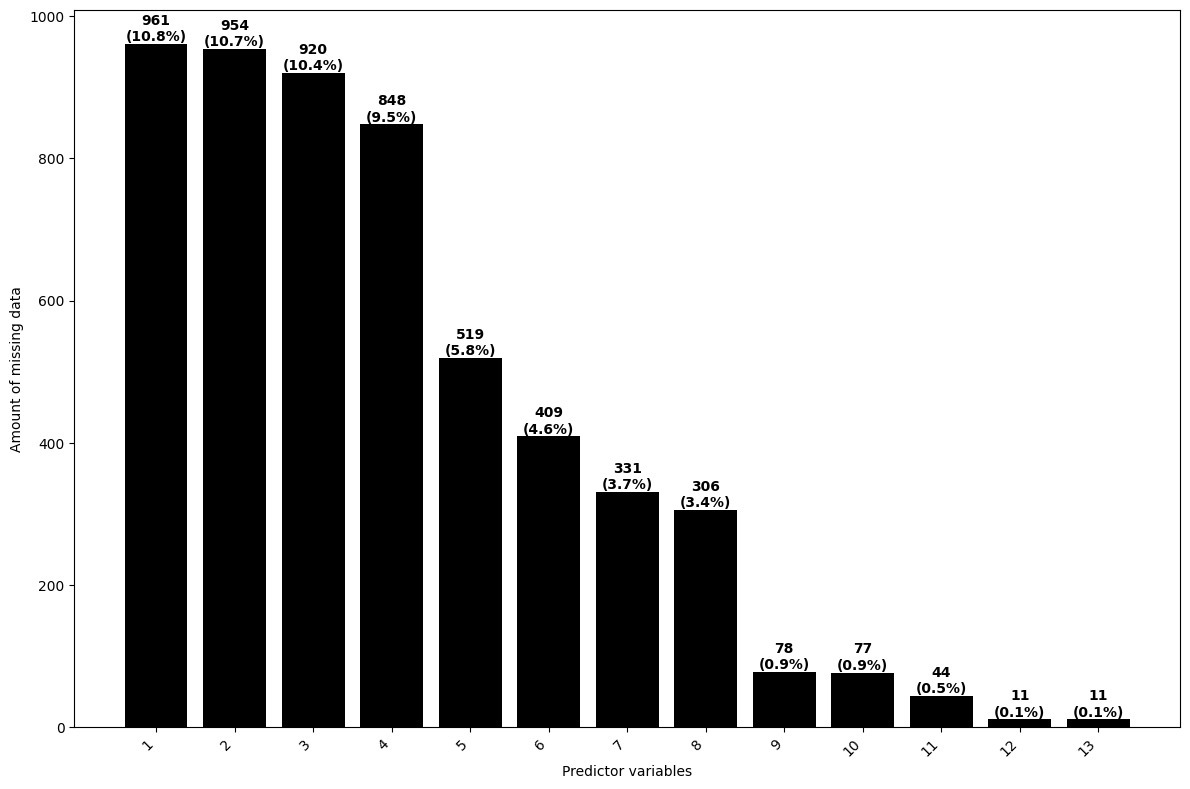


Elbow method for determining the ideal number (k) for multiple imputation with the K-Nearest Neighbors (KNN) algorithm.**~~
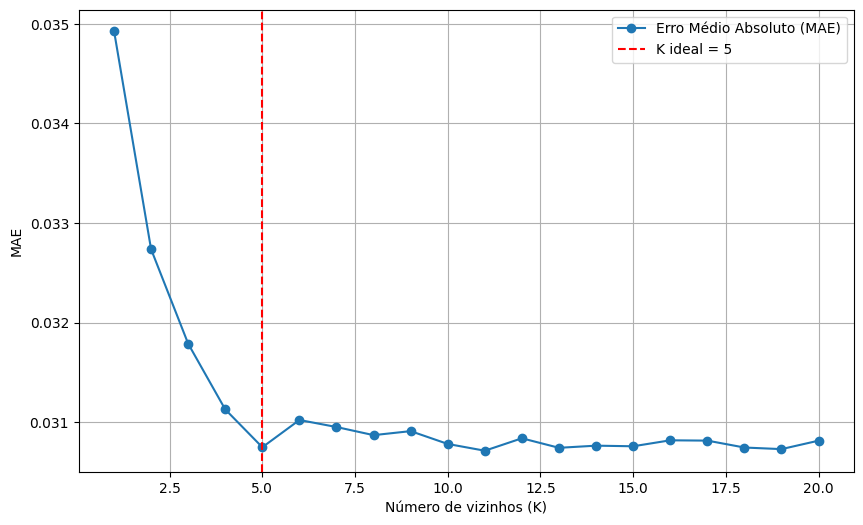
~~**
