# Supplementary material for: Construction of a classification model for dementia among Brazilian adults aged 50 and over
Source: Front Aging Neurosci. 2026 Apr 15;18:1789012. doi: 10.3389/fnagi.2026.1789012 (PMC13126550; doi:10.3389/fnagi.2026.1789012)
Supplement: Supplementary Table 1 — Neuropsychological test battery. [file Table_1.docx]

# Supplementary Table 1 - Neuropsychological Test Battery

| **Cognition (Global Cognition)** | | |
| --- | --- | --- |
| **Time orientation**  Next, I will ask some more questions, still about your memory and concentration. Some questions may seem easy, but others are more difficult. These questions are designed so that no one can answer all of them correctly. Try to answer as best as you can. Interviewer: before starting the tests, make sure the interviewee is wearing glasses, if needed. | | |
| **Variables (name in the database)** | **Variable description** | **Code and description** |
| q5 | Could you tell me today's date? Please provide the day, month, and year. Interviewer's note: Do not give the interviewee the current date; let the interviewee provide it themselves. | \|__\|__\|day  \|__\|__\| month  \|__\|__\|__\|__\| year  (9) Don't know  (10) Not applicable |
| q6 | What day of the week is it? | (1) Sunday  (2) Monday  (3) Tuesday  (4) Wednesday  (5) Thursday  (6) Friday  (7) Saturday  (9) Don't know  (10) No response |
| q7 | Attention Interviewer: Do not read the question aloud to the interviewee. Simply confirm that the previous answers are correct. Is the date correct? | (0) No  (1) Yes |
| q8 | Attention Interviewer: Do not read the question aloud to the interviewee. Simply confirm that the previous answers are correct. Is the month correct? | (0) No  (1) Yes |
| q9 | Attention Interviewer: Do not read the question aloud to the interviewee. Simply confirm that the previous answers are correct. Is the year correct? | (0) No  (1) Yes |
| q10 | Attention Interviewer: Do not read the question aloud to the interviewee. Simply confirm that the previous answers are correct. Is the day of the week correct? | (0) No  (1) Yes |
| **List of 10 words for immediate recall.**  10-word list test  Now I'm going to read a list of 10 words and then ask you to repeat the words you remember. The list is intentionally long to make it difficult for anyone to remember all the words. Most people only remember a few words. Please pay close attention to the list of 10 words, because I cannot repeat them. When I finish, I will ask you to repeat aloud all the words you remember, regardless of the order. Is it clear what we are going to do? Interviewer: if the participant did not understand, explain the task again.  Shall we begin?  Interviewer: read the words to the interviewee, waiting two seconds between each word. | | |
| q13 | Now, when I ask, please tell me the words you can remember. You may begin (start the timer). Interviewer: Start the timer and ask the interviewee to finish the task when the time is up. The maximum time for the task is 2 minutes. On the interviewee's form, mark the list that was read and check the correct words that were remembered. Fill in the answer field with the number of words remembered correctly. | \|__\|__\| number of words remembered  (10) None  (88) maximum |
| **Semantic verbal fluency**  Now I ask that you name several different animals that you can remember. Try to remember as many animals as possible. You will have 1 (one) minute to name these animals. I will tell you when to start.  Interviewer: only if the participant asks for clarification, explaining that animals include birds, insects, fish, etc. | | |
| q14 | Let's begin! (start the timer) Interviewer: If the interviewee remains still, ask "Can you remember any other animals?" Interviewer: Write down all the animals mentioned in the space provided on the pad. Interviewer: Note the number of animals correctly mentioned. | \|__\|__\| number of animals |
| **prospective memory**  I'll give you this sheet of paper and a pencil in a moment. When you receive the clipboard, you should write your initials in the upper left corner of the sheet of paper.  Please write your first and last initials. Is it clear what you need to do?  Interviewer: If it wasn't clear, please explain further.  For those who don't know how to write a note, the instruction will be to mark an "X" in the upper left corner of the sheet of paper. | | |
| q16 | Interviewer: What did the interviewee do when you gave them the paper and pencil? | (0) Wrote the initials of the name/marked an “X” in the upper left corner of the paper  (1) Wrote the initials of the name/marked an “X” elsewhere on the paper  (2) He wrote something else in the upper left corner of the paper.  (3) He did something else  (4) He did nothing |
| **List of 10 words for delayed recall**  A few minutes ago , the computer read/I read a list of words to you, which you repeated. Could you tell me which of those words you remember? I'll tell you when to start. | | |
| q17 | Now, please tell me the words you can remember (start the timer). Interviewer: Start the timer and ask the interviewee to finish the task when the time is up. The maximum time for the task is 2 minutes. On the interviewee's form, mark the list that was read and check the correct words that were remembered. Fill in the answer field with the number of words remembered correctly. | (0) None  \|__\|\|__\| number of words remembered  (10) Maximum |
| **Semantic memory (2 questions about common items and 2 questions about political knowledge)**  Finally, I'm going to ask you the purpose of some objects and the names of some people. | | |
| q18 | What do people usually use to cut paper? | (1) Scissors  (2) Incorrect  (9) Don't know  (10) No response |
| q19 | What is the plant with long, green leaves that produces a long, yellow fruit (when ripe) that we peel to eat? | (1) Banana or banana tree  (2) Incorrect  (9) Don't know  (10) No response |
| q20 | Who is the current president of Brazil?  Interviewer: accept first name or last name or both. | (1) Dilma Rousseff, correct  (2) Incorrect  (9) Don't know  (10) No response |
| q21 | Who is the Vice President of Brazil?  Interviewer: accept first name or last name or both. | (1) Michel Temer, correct  (2) Incorrect  (9) Don't know  (10) No response |
