# Supplementary material for: Construction of a classification model for dementia among Brazilian adults aged 50 and over
Source: Front Aging Neurosci. 2026 Apr 15;18:1789012. doi: 10.3389/fnagi.2026.1789012 (PMC13126550; doi:10.3389/fnagi.2026.1789012)
Supplement: Supplementary Table 2 — Informant Questionnaire on Cognitive Decline in Older Adults (IQCODE), short version with 16 items. [file Table_2.docx]

# Supplementary Table 2 - Informant Questionnaire on Cognitive Decline in Older Adults (IQCODE) short version with 16 items

| **Variables (name in the database)** | **Variable description** | **Code and description** |
| --- | --- | --- |
| iq-1 | Remembering things about family and friends, e.g., occupations, birthdays, addresses. | (1) It has improved a lot  (2) Has there been any improvement? (2)  (3) It didn't change much  (4) Has there been any worsening?  (5) It got much worse  Empty = NaN |
| iq-2 | Remembering things that happened recently | (1) It has improved a lot  (2) Has there been any improvement? (2)  (3) It didn't change much  (4) Has there been any worsening?  (5) It got much worse  Empty = NaN |
| iq-3 | Remember what you talked about in the last few days. | (1) It has improved a lot  (2) Has there been any improvement? (2)  (3) It didn't change much  (4) Has there been any worsening?  (5) It got much worse  Empty = NaN |
| iq-4 | Remember your address and phone number. | (1) It has improved a lot  (2) Has there been any improvement? (2)  (3) It didn't change much  (4) Has there been any worsening?  (5) It got much worse  Empty = NaN |
| iq-5 | Remember the current day and month. | (1) It has improved a lot  (2) Has there been any improvement? (2)  (3) It didn't change much  (4) Has there been any worsening?  (5) It got much worse  Empty = NaN |
| iq-6 | Remember where things are usually stored. | (1) It has improved a lot  (2) Has there been any improvement? (2)  (3) It didn't change much  (4) Has there been any worsening?  (5) It got much worse  Empty = NaN |
| iq-7 | Remembering where things were stored that were  placed in locations different from the usual | (1) It has improved a lot  (2) Has there been any improvement? (2)  (3) It didn't change much  (4) Has there been any worsening?  (5) It got much worse  Empty = NaN |
| iq-8 | Knowing how household appliances work | (1) It has improved a lot  (2) Has there been any improvement? (2)  (3) It didn't change much  (4) Has there been any worsening?  (5) It got much worse  Empty = NaN |
| iq-9 | Learn how to use new household appliances. | (1) It has improved a lot  (2) Has there been any improvement? (2)  (3) It didn't change much  (4) Has there been any worsening?  (5) It got much worse  Empty = NaN |
| iq-10 | Learning new things in general | (1) It has improved a lot  (2) Has there been any improvement? (2)  (3) It didn't change much  (4) Has there been any worsening?  (5) It got much worse  Empty = NaN |
| iq-11 | Following a story in a book or on television | (1) It has improved a lot  (2) Has there been any improvement? (2)  (3) It didn't change much  (4) Has there been any worsening?  (5) It got much worse  Empty = NaN |
| iq-12 | Making decisions about everyday problems. | (1) It has improved a lot  (2) Has there been any improvement? (2)  (3) It didn't change much  (4) Has there been any worsening?  (5) It got much worse  Empty = NaN |
| iq-13 | Handling money for purchases | (1) It has improved a lot  (2) Has there been any improvement? (2)  (3) It didn't change much  (4) Has there been any worsening?  (5) It got much worse  Empty = NaN |
| iq-14 | Dealing with financial issues, such as pensions and banking matters. | (1) It has improved a lot  (2) Has there been any improvement? (2)  (3) It didn't change much  (4) Has there been any worsening?  (5) It got much worse  Empty = NaN |
| iq-15 | Dealing with other everyday mathematical problems, such as knowing how much food to buy, or how much time has passed between visits from family and friends. | (1) It has improved a lot  (2) Has there been any improvement? (2)  (3) It didn't change much  (4) Has there been any worsening?  (5) It got much worse  Empty = NaN |
| iq-16 | Using your intelligence to understand the meaning of things. | (1) It has improved a lot  (2) Has there been any improvement? (2)  (3) It didn't change much  (4) Has there been any worsening?  (5) It got much worse  Empty = NaN |
