# Supplementary material for: Construction of a classification model for dementia among Brazilian adults aged 50 and over
Source: Front Aging Neurosci. 2026 Apr 15;18:1789012. doi: 10.3389/fnagi.2026.1789012 (PMC13126550; doi:10.3389/fnagi.2026.1789012)
Supplement: Supplementary Table 3 — Variables used for subsample selection. [file Table_3.docx]

# Supplementary Table 3 - Variables used for subsample selection

| **Variables (name in the database)** | **Variable description** | **Code and description** |
| --- | --- | --- |
| age | Age of participants | \|__\|__\| years |
| sex | Sex of the participants | (1) Male  (0) Female |
| e22 | What was the last year of school that you were approved for?  If the answer is not spontaneous, read the alternatives to the interviewee.  When the interviewee states that they have completed higher education, ask if they have completed a specialization, master's degree, or doctorate if they do not answer spontaneously. | (1) Never studied  (2) 1st year of primary school  (3) 2nd year of the 1st grade  (4) 3rd year of the 1st grade  (5) 4th grade of the 1st level (former primary or group)  (6) 5th grade of the 1st level  (7) 6th grade of the 1st level  (8) 7th grade of the 1st level  (9) 8th grade of the 1st level (former gymnasium)  (10) 1st year of high school  (11) 2nd year of high school  (12) 3rd year of secondary school (former high school: classical, scientific, normal)  (13) Supplementary/maturity  (14) Incomplete higher education  (15) Completed higher education  (16) Medical specialization/residency  (17) Master's degree  (18) Doctorate  (99) Don't know/no answer = Empty “NaN” |
| **Vision**  **Visual deficits that could affect test performance.** | | |
| n6 | **How would you rate your distance vision (EVEN WHEN WEARING GLASSES OR CONTACT LENSES)? In other words, how would you recognize an acquaintance across the street from about 20 meters away? Read the options to the interviewee.** | (1) Very good or excellent  (2) Good  (3) Regular  (4) Bad  (5) Very bad  (9) Don't know/no answer |
| n7 | **How would you rate your near vision (EVEN WHEN WEARING GLASSES OR CONTACT LENSES)? That is, recognizing an object within reach or reading a newspaper? Read the options to the interviewee.** | (1) Very good or excellent  (2) Good  (3) Regular  (4) Bad  (5) Very bad  (9) Don't know/no answer |
| **Hearing**  **Hearing impairments that could affect test performance.** | | |
| n16 | **How would you rate your hearing (even while using a hearing aid)?** | (1) Very good or excellent  (2) Good  (3) Regular  (4) Bad  (5) Very bad  (9) Don't know/no answer |
| **History of stroke (self-report)** | | |
| n52 | **Has any doctor ever told you that you've had a stroke?** | (0) No (GO TO n54)  (1) Yes  (9) Don't know/no answer (GO TO n54) |
| **History of depression diagnosis (self-report)** | | |
| n59 | **Has any doctor ever told you that you have depression?** | (0) No  (1) Yes  (9) Don't know/no answer |
| **History of Alzheimer's disease diagnosis (self-report)** | | |
| n63 | **Has any doctor ever told you that you have Alzheimer's disease?** | (0) No  (1) Yes  (9) Don't know/no answer |
| **History of Parkinson's disease diagnosis (self-report)** | | |
| n62 | **Has any doctor ever told you that you have Parkinson's disease?** | (0) No  (1) Yes  (9) Don't know/no answer |
| **Clinical symptoms of depression** | | |
| CES-D8 | Clinical symptoms of depression according to the Center for Epidemiology Depression Scale; CESD-8 | Cutoff point: 4 points |
| **Excessive alcohol consumption**  Based on the criteria of the National Institute on Alcohol Abuse and Alcoholism (weekly use of 14 drinks or daily use of 4 drinks for men and weekly use of 7 drinks or daily use of 3 drinks for women) | | |
| l25 | **How many days a week do you usually drink alcohol?** | \|__\| days per week (fill in from 1 to 7 days)  (0) Less than one day a week  (9) Don't know/no answer |
| l26 | **In general, ON THE DAY THAT YOU DRINK , how many alcoholic drinks do you consume?**  **Consider that 1 serving of alcoholic beverage is equivalent to 1 can of beer, 1 glass of wine, or 1 shot of cachaça, whiskey, or any distilled spirit.** | \|__\|__\| doses per day  (99) Don't know/no answer |
| cons_exc_alc | **Weekly use of 14 doses OR daily use of 4 doses for men; weekly use of 7 doses OR daily use of 3 doses for women.** | (1) Excessive consumption  (0) Not excessive  Empty |
| **Instrumental Activities of Daily Living (IADLs)** | | |
| p22 | **Do you have difficulty managing your own money?**  **Managing your own money: having control over your financial resources and carrying out simple economic transactions such as using money to buy food, medicine or personal items; using bank cards and ATMs; and using checks.** | (1) No difficulty (performs the activity effortlessly) (GO TO p24)  (2) Has slight difficulty (only performs the activity with some effort)  (3) Has great difficulty (only performs the activity with great effort, but can do it alone)  (4) Cannot (can only do the activity with the help of another person)  (9) Don't know/no answer (GO TO p24) |
| p24 | **Do you have difficulty using any type of transportation?**  **Use of transportation to travel as a passenger: being transported in a car, taxi, bus, subway, or animal-drawn vehicle, etc.** | (1) No difficulty (performs the activity effortlessly) (GO TO p26)  (2) Has slight difficulty (only performs the activity with some effort)  (3) Has great difficulty (only performs the activity with great effort, but can do it alone)  (4) Cannot (can only do the activity with the help of another person)  (9) Don't know/no answer (GO TO p26) |
| p28 | **Do you have difficulty using the telephone (landline or mobile)?** | (1) No difficulty (performs the activity effortlessly) (GO TO p30)  (2) Has slight difficulty (only performs the activity with some effort)  (3) Has great difficulty (only performs the activity with great effort, but can do it alone)  (4) Cannot (can only do the activity with the help of another person)  (9) Don't know/no answer (GO TO p30) |
| p30 | **Do you have difficulty managing your own medications?**  **Administering your own medications: remembering medication times, picking up the correct doses, removing them from the packaging, and taking them appropriately, as prescribed.** | (1) No difficulty (performs the activity effortlessly) (GO TO p33)  (2) Has slight difficulty (only performs the activity with some effort)  (3) Has great difficulty (only performs the activity with great effort, but can do it alone)  (4) Cannot (can only do the activity with the help of another person)  (9) Don't know/no answer (GO TO p33) |
| **Self-reported or informant-based memory complaints** | | |
| q3 | **Currently, how would you rate your memory?**  **Read the options to the interviewee.** | (1) Excellent  (2) Very good  (3) Good  (4) Regular  (5) Bad  (9) Don't know  (10) No response |
| qp1 | **The purpose of the following questions is to assess the memory and some behaviors of Mr./Ms. (interviewee's name). I would like to know how you would rate Mr./Ms. (interviewee's name)'s memory CURRENTLY?** | (1) Excellent  (2) Very good  (3) Good  (4) Reasonable  (5) Bad  (9) Don't know/no answer |
