# Supplementary material for: Construction of a classification model for dementia among Brazilian adults aged 50 and over
Source: Front Aging Neurosci. 2026 Apr 15;18:1789012. doi: 10.3389/fnagi.2026.1789012 (PMC13126550; doi:10.3389/fnagi.2026.1789012)
Supplement: Supplementary Table 4 — Absolute hand grip strength. [file Table_4.docx]

Supplementary Table 4. Absolute handgrip strength in kilograms-force (kgf) for men and women.

| Absolute handgrip strength in kilograms-force (kgf) for men aged 50 and over. | | | | | |
| --- | --- | --- | --- | --- | --- |
| Age range (years) | **Low Power** | **A little low** | **Moderate** | **A little high** | **High** |
| 50-54 | <37.4 | 37.4 - 43.5 | 43.5 - 48.8 | 48.8 - 54.8 | >54.8 |
| 55-59 | <35.6 | 35.6 - 41.7 | 41.7 - 46.8 | 46.8 - 52.9 | >52.9 |
| 60-64 | <33.6 | 33.6 - 39.7 | 39.7 - 44.7 | 44.7 - 50.4 | >50.4 |
| 65-69 | <31.5 | 31.5 - 37.5 | 37.5 - 42.8 | 42.8 - 48.8 | >48.8 |
| 70-74 | <29.3 | 29.3 - 35.1 | 35.1 - 40.3 | 40.3 - 46.3 | >46.3 |
| 75-79 | <27.0 | 27.0 - 32.7 | 32.7 - 37.8 | 37.8 - 43.5 | >43.5 |
| 80-84 | <24.5 | 24.5 - 30.0 | 30.0 - 34.7 | 34.7 - 40.5 | >40.5 |
| 85-89 | <21.9 | 21.9 - 27.3 | 27.3 - 31.8 | 31.8 - 37.4 | >37.4 |
| 90-94 | <19.2 | 19.2 - 24.6 | 24.6 - 28.9 | 28.9 - 34.2 | >34.2 |
| 95-99 | <16.4 | 16.4 - 21.7 | 21.7 - 25.8 | 25.8 - 30.7 | >30.7 |
| 100+ | <13.5 | 13.5 - 18.7 | 18.7 - 22.7 | 22.7 - 27.2 | >27.2 |
| Absolute handgrip strength in kilograms-force (kgf) for women aged 50 and over. | | | | | |
| Age range (years) | **Low Power** | **A little low** | **Moderate** | **A little high** | **High** |
| 50-54 | <22.4 | 22.4 - 26.4 | 26.4 - 29.4 | 29.4 - 34.0 | >34.0 |
| 55-59 | <21.5 | 21.5 - 25.5 | 25.5 - 28.9 | 28.9 - 33.0 | >33.0 |
| 60-64 | <20.6 | 20.6 - 24.5 | 24.5 - 27.7 | 27.7 - 31.8 | >31.8 |
| 65-69 | <19.5 | 19.5 - 23.4 | 23.4 - 26.5 | 26.5 - 30.4 | >30.4 |
| 70-74 | <18.3 | 18.3 - 22.0 | 22.0 - 25.0 | 25.0 - 28.9 | >28.9 |
| 75-79 | <16.9 | 16.9 - 20.6 | 20.6 - 23.5 | 23.5 - 27.3 | >27.3 |
| 80-84 | <15.5 | 15.5 - 19.0 | 19.0 - 21.9 | 21.9 - 25.5 | >25.5 |
| 85-89 | <14.0 | 14.0 - 17.3 | 17.3 - 20.1 | 20.1 - 23.6 | >23.6 |
| 90-94 | <12.2 | 12.2 - 15.3 | 15.3 - 17.9 | 17.9 - 21.1 | >21.1 |
| 95-99 | <10.3 | 10.3 - 13.3 | 13.3 - 15.7 | 15.7 - 18.5 | >18.5 |
| 100+ | <8.3 | 8.3 - 11.2 | 11.2 - 13.4 | 13.4 - 16.5 | >16.5 |
