# Supplementary material for: Construction of a classification model for dementia among Brazilian adults aged 50 and over
Source: Front Aging Neurosci. 2026 Apr 15;18:1789012. doi: 10.3389/fnagi.2026.1789012 (PMC13126550; doi:10.3389/fnagi.2026.1789012)
Supplement: Supplementary Table 5 — Comparison of the occurrence of cognitive dysfunction between predictors. [file Table_5.docx]

| Supplementary Table 5. Comparison of the occurrence of cognitive dysfunction among the predictors. | | | | | |
| --- | --- | --- | --- | --- | --- |
| **Features** | | **Normal cognition**  **N = 7962 (89.6%)** | | **Insanity**  **N = 845 (9.5%)** | *P* value |
| Sex, n (%)  Men  Women | | 3522 (44.2)  4440 (55.7) | | 312 (37.0)  532 (62.9) | 0.0065 |
| Age, n (%)  50-54  55-59  60-64  65-69  70-74  75-79  80-84  85-89  90+ | | 1927 (24.2)  1499 (18.8)  1302 (16.3)  1028 (12.9)  878 (11.0)  712 (8.9)  393 (4.9)  187 (2.3)  36 (0.4) | | 134 (15.8)  166 (19.6)  155 (18.3)  144 (17.0)  80 (9.4)  48 (5.6)  28 (3.3)  27 (3.2)  63 (7.4) | <0.001 |
| Educational level, n (%)  Illiterate  Less than elementary school  Completed Elementary Education  Incomplete High School Education  Completed High School  Higher Education or More | | 1031 (12.9)  1548 (19.4)  2472 (31.0)  936 (11.7)  1395 (17.5)  580 (7.2) | | 332 (39.2)  217 (25.6)  199 (23.5)  46 (5.4)  28 (3.3)  23 (2.7) | <0.001 |
| Skin color, n (%)  White  Black  Brown  Yellow  Indigenous | | 3157 (40.9)  713 (9.2)  3589 (46.5)  73 (0.9)  183 (2.3) | | 248 (31.3)  114 (14.4)  395 (50.0)  9 (1.1)  24 (3.0) | 0.0029 |
| Occupational situation , n (%)  No  Yes | | 5465 (68.6)  2497 (31.3) | | 691 (81.7)  154 (18.2) | <0.001 |
| Marital status, n (%)  Single  Married/cohabiting/common-law marriage  Divorced or separated  Widower | | 848 (10.6)  4705 (59.0)  978 (12.2)  1431 (17.9) | | 112 (13.2)  410 (48.5)  98 (11.6)  225 (26.6) | 0.0203 |
| **Health and lifestyle habits** | |  | |  |  |
| BMI, n (%)  Severely underweight  Underweight | | 31 (0.4)  109 (1.4) | | 4 (0.5)  26 (3.4) | <0.001 |
| Normal weight  Overweight | | 2110 (27.5)  3097 (40.3) | | 271 (35.8)  257 (33.9) |  |
| Moderately obese (grade I)  Severe obesity (grade II) | | 1628 (21.2)  502 (6.5) | | 142 (18.7)  42 (5.5) |  |
| Morbid obesity (grade III) | | 191 (2.4) | | 14 (1.8) |  |
| PA level, n (%)  High  Moderate  Low | | 2330 (30.9)  2190 (29.1)  3004 (39.9) | | 149 (19.3)  155 (20.1)  467 (60.5) | <0.001 |
| HGS, n (%)  High  A little high  Moderate  A little low  Low | | 254 (3.3)  823 (10.7)  1234 (16.0)  2217 (28.8)  3162 (41.1) | | 11 (1.3)  46 (5.7)  83 (10.4)  171 (21.4)  485 (60.9) | <0.001 |
| Hypertension, n (%)  No  Yes | | 6812 (87.0)  1014 (12.9) | | 698 (85.7)  116 (14.2) | 0.1061 |
| Diabetes, n (%)  No  Yes | | 6669 (84.1)  1259 (15.8) | | 666 (79.5)  171 (20.4) | 0.0061 |
| High cholesterol, n (%)  No  Yes | | 5453 (69.0)  2447 (30.9) | | 554 (66.6)  277 (33.3) | 0.0024 |
| Visual loss, n (%)  Cataract  No  Yes  Retinopathy  No  Yes | | 6797 (93.3)  481 (6.6)  7063 (98.0)  142 (1.9) | | 727 (93.2)  53 (6.7)  634 (96.2)  25 (3.7) | 0.3090  0.0016 |
| Hearing, n (%)  Good  Regular | | 5554 (69.8)  1985 (24.9) | | 525 (62.4)  197 (23.4) | 0.0049 |
| Bad | | 416 (5.2) | | 119 (14.1) |  |
| Smoking, n (%)  No  Yes | | 416 (5.2)  7539 (94.7) | | 119 (14.1)  722 (85.8) | 0.0012 |
| Excessive alcohol consumption, n (%)  No  Yes | | 7740 (97.7)  179 (2.2) | | 824 (98.2)  15 (1.7) | 0.9147 |
| **Psychosocial** |  | |  | |  |
| Social isolation, n (%)  No  Yes | | 7559 (94.9)  403 (5.0) | | 751 (88.8)  94 (11.1) | <0.001 |
| Loneliness, n (%)  Never  Sometimes  Always | | 3765 (52.0)  2349 (32.4)  1124 (15.5) | | 276 (42.8)  165 (25.6)  203 (31.5) | <0.001 |
| Life satisfaction, median (IQR) | | 8.0 (5.0-10.0) | | 7.0 (4.0-10.0) | <0.001 |
| Depressive symptoms, n (%)  No  Yes | | 4904 (66.8)  2434 (33.1) | | 302 (45.9)  355 (54.0) | <0.001 |
| Legend: ELSI-Brazil: Longitudinal Study of the Health of Brazilian Elderly; SD: Standard Deviation; BMI: Body Mass Index; PA: Physical Activity; HGS: Handgrip Strength; IQR: Interquartile Range. | | | | | |
