# Supplementary material for: Construction of a classification model for dementia among Brazilian adults aged 50 and over
Source: Front Aging Neurosci. 2026 Apr 15;18:1789012. doi: 10.3389/fnagi.2026.1789012 (PMC13126550; doi:10.3389/fnagi.2026.1789012)
Supplement: Supplementary Table 6 — Random forest analysis using the out-of-bag (OOB) error curve. [file Table_6.docx]

| Supplementary Table 6. Random Forest analysis using the Out-Of-Bag (OOB) graded error curve. | | | |
| --- | --- | --- | --- |
| **k** | **features** | **oob_error_mean** | **oob_error_std** |
| 1 | ['educ'] | 0.316099279 | 0.002003297 |
| 2 | ['educ', 'age'] | 0.223274375 | 0.012806434 |
| 3 | ['educ', 'age', 'satis_vid'] | 0.227137278 | 0.010755953 |
| 4 | ['educ', 'age', 'satis_vid', 'BMI'] | 0.17430384 | 0.005020587 |
| 5 | ['educ', 'age', 'satis_vid', 'BMI', 'HGS'] | 0.146268017 | 0.003125541 |
| 6 | ['educ', 'age', 'satis_vid', 'BMI', 'HGS', 'e7'] | 0.11792695 | 0.002240108 |
| 7 | ['educ', 'age', 'satis_vid', 'BMI', 'HGS', 'e7', 'skin_color'] | 0.10405014 | 0.001576472 |
| 8 | ['educ', 'age', 'satis_vid', 'BMI', 'HGS', 'e7', 'skin_color', 'IPAQ'] | 0.091202671 | 0.001161927 |
| 9 | ['educ', 'age', 'satis_vid', 'BMI', 'HGS', 'e7', 'skin_color', 'IPAQ', ‘lonel’] | 0.087431202 | 0.001083087 |
| 10 | ['educ', 'age', 'satis_vid', 'BMI', 'HGS', 'e7', 'skin_color', 'IPAQ', ‘lonel’, 'hearing'] | 0.084931232 | 0.000936295 |
| 11 | ['educ', 'age', 'satis_vid', 'BMI', 'HGS', 'e7', 'skin_color', 'IPAQ', ‘lonel’, 'hearing', 'CES_D8'] | 0.083437024 | 0.000885907 |
| 12 | ['educ', 'age', 'satis_vid', 'BMI', 'HGS', 'e7', 'skin_color', 'IPAQ', ‘lonel’, 'hearing', 'CES_D8', 'colest'] | 0.08244894 | 0.000700413 |
| 13 | ['educ', 'age', 'satis_vid', 'IMC', 'HGS', 'e7', 'skin_color', 'IPAQ', ‘lonel’, 'hearing', 'CES_D8', 'colest', 'sex'] | 0.082311065 | 0.000660359 |
| 14 | ['educ', 'age', 'satis_vid', 'BMI', 'HGS', 'e7', 'skin_color', 'IPAQ', ‘lonel’, 'hearing', 'CES_D8', 'colest', 'sex', 'occup'] | 0.081711852 | 0.000663515 |
| **15** | **['educ', 'age', 'satis_vid', 'BMI',** 'HGS'**, 'e7',** 'skin_color'**, 'IPAQ',** ‘lonel’**, 'hearing', 'CES_D8', 'colest', 'sex',** 'occup'**, 'diabetes']** | **0.081240494** | **0.000606589** |
| 16 | ['educ', 'age', 'satis_vid', 'BMI', 'HGS', 'e7', 'skin_color', 'IPAQ', ‘lonel’, 'hearing', 'CES_D8', 'colest', 'sex', 'occup', 'diabetes', 'isol_soc'] | 0.081700069 | 0.000577713 |
| 17 | ['educ', 'age', 'satis_vid', 'BMI', 'HGS', 'e7', 'skin_color', 'IPAQ', ‘lonel’, 'hearing', 'CES_D8', 'colest', 'sex', 'occup', 'diabetes', 'isol_soc', 'tabag'] | 0.08172835 | 0.000618322 |
| 18 | ['educ', 'age', 'satis_vid', 'BMI', 'HGS', 'e7', 'skin_color', 'IPAQ', ‘lonel’, 'hearing', 'CES_D8', 'colest', 'sex', 'occup', 'diabetes', 'isol_soc', 'tabag', 'retinop'] | 0.081661774 | 0.000604278 |
| Source: From the authors  Legend: educ: Educational level; satis_vid: Life satisfaction; BMI: Body Mass Index; HGS: Handgrip Strength; e7: Marital status; IPAQ: Physical activity level; lonel: loneliness; CES_D8: Depressive symptoms; cholest: High cholesterol; 'occup': Occupational status; isol_soc: Social isolation; tabag: Smoking; retinop: Retinopathy | | | |
