# Supplementary material for: Construction of a classification model for dementia among Brazilian adults aged 50 and over
Source: Front Aging Neurosci. 2026 Apr 15;18:1789012. doi: 10.3389/fnagi.2026.1789012 (PMC13126550; doi:10.3389/fnagi.2026.1789012)
Supplement: Supplementary Table 7 — Variables and their respective values. [file Table_7.docx]

| Supplementary Table 7. Variables and their respective values | |
| --- | --- |
| Independent variables | Values assigned to categories |
| Educational level | 1=Illiterate, 2=Less than Elementary School, 3=Completed Elementary School, 4=Incomplete High School, 5=Completed High School, 6=Higher Education or More |
| Age | 50-54=1, 55-59=2, 60-64=3, 65-69=4, 70-74=5, 75-79=6, 80-84=7, 85-89=8, 90+=9 |
| Life satisfaction | Step number indicated = 0 to 10 |
| BMI | 1=Severely underweight, 2=Underweight, 3=Normal weight, 4=Overweight, 5=Moderately obese (grade I), 6=Severely obese (grade II), 7=Morbidly obese (grade III) |
| HGS | 1=High, 2=Slightly High, 3=Moderate, 4=Slightly Low, 5=Low Strength |
| Marital status | 1=Single, 2=Married/cohabiting/common-law marriage, 3=Divorced or separated, 4=Widowed |
| Skin color | 1=White, 2=Black, 3=Brown, 4=Yellow (of Asian origin, Japanese, Chinese, Korean, etc.), 5=Indigenous |
| Level of physical activity | 1=High, 2=Moderate, 3=Low |
| Loneliness | 1=Never, 2=Sometimes, 3=Always |
| Hearing | 1=Good, 2=Average, 3=Poor |
| Depressive symptoms | 0=No, 1=Yes |
| Sex | 1=Male, 0=Female |
| High cholesterol | 0=No, 1=Yes |
| Occupational status | 0=No, 1=Yes |
| Diabetes | 0=No, 1=Yes |

Legend: BMI: Body Mass Index; HGS: Handgrip Strength.
